# Anti-Proliferative Activity of Ethylenediurea Derivatives with Alkyl and Oxygen-Containing Groups as Substituents

**DOI:** 10.3390/biomedicines13020316

**Published:** 2025-01-29

**Authors:** Maxim Oshchepkov, Leonid Kovalenko, Antonida Kalistratova, Galina Sherstyanykh, Evgenia Gorbacheva, Alexey Antonov, Nisreen Khadour, Mikhail Akimov

**Affiliations:** 1 Faculty of Chemico-Pharmaceutical Technologies and Biomedical Drugs, Mendeleev University of Chemical Technology of Russia, Miusskaya sq. 9, 125047 Moscow, Russia; lkovalenko@muctr.ru (L.K.); kalistratova.a.v@muctr.ru (A.K.); 2 Shemyakin-Ovchinnikov Institute of Bioorganic Chemistry, Russian Academy of Sciences, Ul. Miklukho-Maklaya, 16/10, 117997 Moscow, Russia; galya24may@gmail.com (G.S.); gorevig2000@gmail.com (E.G.); nisreenkhadour75@gmail.com (N.K.); 3 Faculty of Mechanics and Mathematics, Moscow State University, Leninskiye Gory, 1, 119234 Moscow, Russia; alexey.p.antonov@gmail.com; 4 Moscow Center for Advanced Studies, Kulakova Str. 20, 123592 Moscow, Russia

**Keywords:** carbamates, oxamates, synthetic cytokinins, ethylene diurea cytokininss, anti-proliferation, temozolomide, doxorubicin, glioblastoma, melanoma, breast cancer

## Abstract

**Background/Objectives**: Natural cytokinins are a promising group of anti-tumor agents. In this work, we hypothesized that modification of the ethylenediurea moiety with alkyl and oxygen-containing groups could be a way to enhance the anti-proliferative properties of the molecule. **Methods**: Ten new analogs of ethylenediurea with these substitutions were tested for anti-proliferative activity in the human cancer cell lines MDA-MB-231 (breast cancer), A-375 (melanoma), and U-87 MG (glioblastoma) during 72 h of incubation using resazurin test and evaluated the substances receptor using molecular docking. **Results**: The compound with the carbamate link and ethylene substituent on the phenyl ring inhibited proliferation in these models by 70–90% without cytotoxic effects. The compound did not affect the viability of the immortalized fibroblast cell line Bj-5ta. The compound was also able to enhance the action of doxorubicin and temozolomide by about 20%. According to the molecular modeling data, the probable receptor target for the synthesized compound was the A2AR adenosine receptor. **Conclusions**: The results obtained on the ethylenediurea analogs with ethyl substituent in the aromatic ring are promising for the development of novel anticancer therapeutics.

## 1. Introduction

Although generally known as plant growth regulators [1,2], synthetic derivatives of phytohormones of the cytokinin family are promising substances with anticancer [3,4], anti-proliferative [5,6], and immunomodulatory activity [4,7]. The most widely known chemical modifications of cytokinin molecules are adducts with ribose residue [3,8,9,10], haloid introduction into various parts of the molecule [11], and synthetic analogs of the ethylenediurea family [6,12,13].

**Figure 1:**
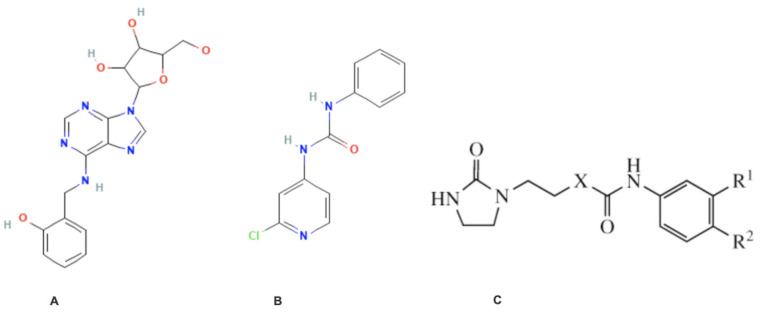
The structures of ortho-tropolin riboside (**A**), forchlorfenuron (**B**), and ethylenediurea-based aryl carbamate variant (**C**).

Ribosides of adenine cytokinins exhibit varying degrees of cytotoxic properties against a wide range of malignant human tumor cells, including glioblastoma, rhabdomyosarcoma, melanoma, breast cancer, leukemia, colon cancer, lung cancer, tumors of the central nervous system, and prostate, ovarian and kidney cancers [3,8,9,10]. Ortho-topolin riboside (Figure 1) shows high activity (IC_50_ = 0.5–11.6 μM) against several human malignant cell lines. Although the cytotoxic activity of the ribosides trans-Zeatin (tZ), N6-(2-Isopentenyl)adenine (iP), and Benzyl aminopurine (BA) against tumor cells was demonstrated as early as the end of the 1960s to the middle of the 1970s, these data were not introduced into medical practice because of the indiscriminate effect on malignant and healthy cells [3,9,14].

Forchlorfenuron (CPPU, Figure 1) is a synthetic chlorinated cytokinin analog [11]. It inhibits proliferation, migration, and invasion in cells of various types of oncological diseases, such as tumors of the prostate, lung, colon, breast, ovary, and cervix and mesothelioma. CPPU has been found to inhibit tumor growth in mouse trials [15]. Forchlorfenuron derivatives with oxygen- and sulfur-containing groups at significantly lower doses (4–33 μM versus 100 μM for CPPU) were found to inhibit the proliferation of cancer cells in several endometrial and ovarian cell lines [11].

Ethylenediurea (EDU, Figure 1) synthetic analogs of cytokinins with aryl carbamate and aryl urea components and chlorine substitutions display anti-proliferative activity without cytotoxicity in MDA-MB-231, A-375, and U-87 MG cell lines [6,12,13], presumably via the adenosine A2 receptor and CDK2 [6]. A2AR has emerged as a promising target for cancer immunotherapy. Recent studies and clinical trials have focused on targeting the A2AR pathway to improve cancer treatment outcomes. A2AR antagonists, such as NIR178 and Ciforadenant, have been investigated for their potential to enhance anti-tumor immunity and are being tested in combination with other immunotherapies [16]. The combination of A2AR inhibitors with other treatments, such as anti-PD1 therapies, has shown potent anti-tumor effects in animal models [17]. Recent clinical trials have focused on the use of A2AR antagonists in treatment-refractory cancers, such as renal cell carcinoma, with promising early results [18]. The aberrant activation of CDK2, often due to high levels of its regulatory cyclins A and E, also leads to uncontrolled cell proliferation, a hallmark of cancer [19]. In addition, compounds with pure anti-proliferative activity may represent a way to overcome resistance to traditional chemotherapies.

Several lines of EDU modification are possible: the variations of the adducts on both sides of the ethylenediurea, the variation of the carbamate and urea links, haloid adducts, and the addition of oxygen-containing and alkyl groups. Data from our previous research [6,13] indicate that EDU modification with an imidazole moiety may affect cell proliferation without a cytotoxicity component, and the variants of the chemical link could be a modulating factor of the substance’s activity. In addition, the size and polarity of the substituent groups in the aryl moiety substantially affect the substance’s activity. At the same time, chlorine substituents usually render the molecule cytotoxic. Thus, we hypothesized that the modification of the imidazoline-based EDU analog with alkyl and oxygen-containing groups could be a way to enhance the molecule’s anti-proliferative properties. In this work, ten novel EDU derivatives with the hypothesized alkyl and oxygen-containing groups as substituents at various positions in the aromatic moiety were synthesized and tested for anti-proliferative and cytotoxic activity in MDA-MB-231, U-87 MG, and A-375 cell lines. Two of the compounds displayed selective anti-proliferative activity towards breast cancer, melanoma, and glioblastoma cell lines. The leading compound was able to enhance the activity of doxorubicin and temozolomide by 20–30%. Based on the molecular docking data, the mechanism of action was the probable inhibition of the A2AR receptor.

## 2. Materials and Methods

### 2.1. Materials

L-glutamine, fetal bovine serum, penicillin, streptomycin, amphotericin B, Hanks’ salts, Earle’s salts, trypsin, DMEM, MEM, and (4,5-dimethylthiazol-2-yl)-2,5-diphenyltetrazolium bromide (MTT) were purchased from PanEco, Moscow, Russia. Isopropanol, HCl, CHAPS, protease inhibitor cocktail, EDTA, dithiothreitol, HEPES, DMSO, toluene, INT, diaphorase, NAD^+^, acetonitrile, carbon tetrachloride, diethylenetriamine, urea, triethylamine, 4-chlorophenyl isocyanate, 3,4-dichlorophenyl isocyanate, and D-glucose were purchased from Sigma-Aldrich, St. Louis, MO, USA.

Human metastatic breast cancer cell line MDA-MB-231 (ATCC HTB-26), immortalized human fibroblasts Bj-5ta (ATCC CRL-4001), human embryonic kidney cell line HEK 293 (ATCC CRL-1573), human melanoma cell line A-375 (ATCC CRL-1619), human glioblastoma cell line U-87 MG (ATCC HTB-14), and human neuroblastoma cell line SH-SY5Y (ATCC CRL-2266) were purchased from ATCC, Manassas, VA, USA.

### 2.2. Chemical Synthesis

The general procedure for the synthesis of aryl ureas is described in the literature [6,13]. The urea compounds used in this paper were simultaneously tested for plant growth-regulating activity, and their synthesis was reported separately [20].

1-(2-Aminoethyl)-2-imidazolidinone for the synthesis of aryl ureas was obtained through the condensation of diethylene triamine with urea in 65% yield according to the procedure presented in the work [13]. First, 38.6 g (0.375 mol) of diethylene triamine was poured into a three-neck flask equipped with a Dean–Stark apparatus, a reflux condenser, a thermometer, and a magnetic stirrer and then 40% aqueous urea solution was added, containing 15.0 g (0.25 mol) of urea. The mixture was heated for 1 h at a temperature of 100–125 °C with the gradual distillation of water. Then, the temperature was gradually increased as follows: another 90 min at 140 °C and another 90 min at 150 °C. Then, the temperature was maintained at 160 °C for 4 h until the cessation of ammonia emission. The product was purified by vacuum distillation. As a result, 20.95 g (65% yield) of a viscous yellowish liquid was obtained, with a crystallization temperature close to room temperature and a boiling point Tbp = 166–170 °C/1 mm Hg. Literature data: Tbp = 138–140 °C/0.15 mm Hg.

Next, 31 mmol of 1-(2-aminoethyl)-2-imidazolidinone, which was obtained through the condensation of diethylene triamine with urea in 65% yield according to the procedure presented in the work [13], in 50 mL of dry toluene was poured into a three-neck flask with a thermometer, a dropping funnel, and a magnetic stirrer. The mixture was cooled in an ice bath to a temperature no higher than 5 °C. Then, a solution of 31 mmol of the relevant phenyl isocyanate in 50 mL of dry toluene was added dropwise with stirring, with the temperature kept no higher than 5 °C. The reaction mixture was stirred overnight. The precipitate was filtered off and purified through crystallization from a suitable solvent or by column chromatography.

The structures of the obtained compounds were simple enough, and the synthesis procedure was straightforward. Thus, we used only HPLC-MS with two decimal places precision and ^1^H-NMR for structural confirmation.

The general procedure of aryl carbamate (2,4,6,9,10) synthesis according to ref. [6,13] was as follows: In a round bottom flask equipped with a calcium chloride tube and magnetic stirrer, 6.55 mmol of 1-(2-hydroxyethyl)-2-imidazolidinon in a small volume of dry acetonitrile, solution of 6.58 mmol of the relevant phenyl isocyanate in dry acetonitrile (total volume 50 mL), and 2–3 drops of triethylamine were stirred overnight. The reaction mixture was evaporated to dryness, and the residue was recrystallized from methanol and isopropanol. The precipitate was filtered off and purified through crystallization from a suitable solvent or using column chromatography.

2-(2-oxoimidazolidin-1-yl)ethyl-N-(2,6-dimethylphenyl) urea (1) 59% yield. Column chromatography with chloroform/isopropanol 9:1 mixture. M.p. = 212–214 °C. ^1^H NMR (DMSO-d_6_, δ, ppm, J, Hz): 2.12 (s, 6H, -CH_3_); 3.07–3.09 (m, 2H); 3.12–3.23 (m, 4H); 3.32–3.37 (m, 2H, -CH_2_-); 6.01 (t, 1H, -CH_2_-NH-C(O)-NH-, J = 5.4); 6.26 (s, 1H, -NH-C(O)-N-); 6.97–7.04 (m, 3H, CHar); 7.50 (s, 1H, -C(O)-NH-Ar). HPLC-MS: [M + 1]^+^ 277.41; calculated value is 277.34.

2-(2-oxoimidazolidin-1-yl)ethyl-N-(p-tolil) urea (3) 31% yield. Recrystallization from isopropanol. M.p. = 189–190 °C. ^1^H NMR (DMSO-d_6_, δ, ppm, J, Hz): 2.19 (s, 3H, -CH_3_); 3.07–3.13 (m, 2H); 3.15–3.20 (m, 2H); 3.20–3.25 (m, 2H); 3.31–3.39 (m, 2H) (-CH_2_-); 5.98 (t, 1H, -CH_2_-NH-C(O)-NH-, J = 5.4); 6.16 (s, 1H, -NH-C(O)-N-); 6.99 (d, 2H, J = 8.2); 7.23 (d, 2H, J = 8.4, CHar); 8.29 (s, 1H, -C(O)-NH-Ar). HPLC-MS: [M + 1]^+^ 263.30; calculated value is 263.31.

2-(2-oxoimidazolidin-1-yl)ethyl-N-(2-ethylphenyl) urea (5) 62% yield. Column chromatography with chloroform/isopropanol 9:1 mixture. M.p. = 148–149 °C. ^1^H NMR (DMSO-d_6_, δ, ppm, J, Hz): 1.10 (t, 3H, -CH_3_, J = 7.5); 2.51 (q, 2H, -CH_2_-, J = 7.9); 3.07–3.12 (m, 2H); 3.16–3.23 (m, 4H); 3.32–3.38 (m, 2H, -CH_2_-); 3.67 (s, 3H, -OCH_3_); 6.30 (s, 1H, -NH-C(O)-N-); 6.51 (t, 1H, -CH_2_-NH-C(O)-NH-, J = 5.4); 6.88–6.93 (m, 1H); 7.04–7.12 (m, 2H, CHar); 7.66 (s, 1H, -C(O)-NH-Ar); 7.68–7.73 (m, 1H, CHar). HPLC-MS: [M + 1]^+^ 277.41; calculated value is 277.34.

2-(2-oxoimidazolidin-1-yl)ethyl-N-(2,4-dimethylphenyl) urea (7) 58% yield. Column chromatography with chloroform/isopropanol 9:1 mixture. M.p. = 178–181 °C. ^1^H NMR (DMSO-d_6_, δ, ppm, J, Hz): 2.11 (s, 3H, -CH_3_); 2.18 (s, 3H, -CH_3_); 3.09–3.12 (m, 2H); 3.16–3.24 (m, 4H); 3.34–3.38 (m, 2H, -CH_2_-); 6.30 (t, 1H, -CH_2_-NH-C(O)-NH-, J = 5.5); 6.16 (s, 1H, -NH-C(O)-N-); 6.84–6.87 (m, 1H); 6.89–6.90 (m, 1H, CHar); 7.51 (s, 1H, -C(O)-NH-Ar); 7.51–7.53 (m, 1H, CHar). HPLC-MS: [M + 1]^+^ 277.40; calculated value is 277.34.

2-(2-oxoimidazolidin-1-yl)ethyl-N-(4-methoxyphenyl) urea (8) 35% yield. Column chromatography with chloroform/isopropanol 9:1 mixture. M.p. = 155–157 °C. ^1^H NMR (DMSO-d_6_, δ, ppm, J, Hz): 3.11–3.17 (m, 2H); 3.17–3.31 (m, 4H); 3.35–3.42 (m, 2H, -CH_2_-); 3.67 (s, 3H, -OCH_3_); 5.93 (t, 1H, -CH_2_-NH-C(O)-NH-, J = 5.4); 6.16 (s, 1H, -NH-C(O)-N-); 6.76–6.87 (m, 2H); 7.24–7.32 (m, 2H, CHar); 8.20 (s, 1H, -C(O)-NH-Ar). HPLC-MS: [M + 1]^+^ 279.38; calculated value is 279.31.

2-(2-oxoimidazolidin-1-yl)ethyl-N-(2,6-dimethylphenyl) carbamate (2) 30% yield. Column chromatography with chloroform/isopropanol 9:1 mixture. M.p. = 147–148 °C. ^1^H NMR (DMSO-d_6_, δ, ppm, J, Hz): 3.18–3.24 (m, 2H); 3.26–3.31 (m, 2H); 3.39–3.45 (m, 2H, -CH_2_-); 4.12 (t, 2H, -CH_2_-O-, J = 5.5); 6.36 (s, 1H, -NH-C(O)-N-); 7.04 (m, 1H, CHar); 8.73 (s, 1H, -C(O)-NH-Ar). HPLC-MS: [M + 1]^+^ 278.37; calculated value is 278.32.

2-(2-oxoimidazolidin-1-yl)ethyl-N-(p-tolil) carbamate (4) 36% yield. Recrystallization from isopropanol. M.p. = 158–160 °C. ^1^H NMR (DMSO-d_6_, δ, ppm, J, Hz): 2.22 (s, 3H, -CH_3_); 3.14–3.25 (m, 2H); 3.33 (t, 2H, J = 5.6); 3.36–3.45 (m, 2H); 4.14 (t, 2H, -CH_2_-, J = 5.6); 6.21 (s, 1H, -NH-C(O)-N-); 6.96–7.17 (m, 2H); 7.25–7.35 (m, 2H, CHar); 9.38 (s, 1H, -C(O)-NH-Ar). HPLC-MS: [M + 1]^+^ 264.25; calculated value is 264.30.

2-(2-oxoimidazolidin-1-yl)ethyl-N-(2-ethylphenyl) carbamate (6) 50% yield. Column chromatography with chloroform/isopropanol 9:1 mixture. M.p. = 115–117 °C. ^1^H NMR (DMSO-d_6_, δ, ppm, J, Hz): 1.09 (td, 3H, -CH_3_, J = 7.4, 1.9); 2.57 (q, 2H, -CH_2_-, J = 7.5, 1.9); 3.16–3.22 (m, 2H); 3.26–3.29 (m, 2H); 3.31–3.41 (m, 2H, -CH_2_-); 4.09–4.14 (m, 2H, -CH_2_-O-); 6.35 (s, 1H, -NH-C(O)-N-); 7.08–7.15 (m, 2H); 7.16–7.21 (m, 1H); 7.23–7.28 (m, 1H, CHar); 8.85 (s, 1H, -C(O)-NH-Ar). HPLC-MS: [M + 1]^+^ 278.37; calculated value is 278.32.

2-(2-oxoimidazolidin-1-yl)ethyl-N-(4-methoxyphenyl) carbamate (9) 18% yield. Column chromatography with chloroform/isopropanol 9:1 mixture. M.p. = 127–133 °C. ^1^H NMR (DMSO-d_6_, δ, ppm, J, Hz): 3.19–3.22 (m, 2H); 3.28 (t, 2H, J = 5.5); 3.37–3.43 (m, 2H, -CH_2_-); 3.68 (s, 3H, -OCH_3_); 4.12 (t, 2H, -CH_2_-O-, J = 5.5); 6.35 (s, 1H, -NH-C(O)-N-); 6.84 (d, 2H, J = 8.9); 7.34 (d, 2H, CHar, J = 8.0); 9.44 (s, 1H, -C(O)-NH-Ar). HPLC-MS: [M + 1]^+^ 280.38; calculated value is 280.30.

2-(2-oxoimidazolidin-1-yl)ethyl-N-(4-methoxycarbonylphenyl) carbamate (10) 30% yield. Column chromatography with chloroform/isopropanol 9:1 mixture. M.p. = 198–201 °C. ^1^H NMR (DMSO-d_6_, δ, ppm, J, Hz): 3.18–3.23 (m, 2H); 3.33 (t, 2H, J = 5.5); 3.39–3.44 (m, 2H, -CH_2_-); 3.80 (s, 3H, -CH_3_); 4.19 (t, 2H, -CH_2_-O-, J = 5.5); 6.21 (s, 1H, -NH-C(O)-N-); 7.53–7.61 (m, 2H); 7.81–7.92 (m, 1H, CHar); 9.94 (s, 1H, -C(O)-NH-Ar). HPLC-MS: [M + 1]^+^ 308.28; calculated value is 308.31.

### 2.3. Synthesized Compounds’ Characterization

The structures of all synthesized compounds were confirmed with ^1^H NMR spectroscopy and mass spectrometry. The purity of the compounds was confirmed with HPLC-MS and was in the range of 95–99%. ^1^H-spectra were recorded with a “Bruker DRX-400” spectrometer operating at 400.13 MHz frequency, using DMSO-d_6_ as a solvent and TMS as an internal standard. Chemical shifts were measured with 0.01 ppm accuracy, and coupling constants are reported in Hertz. HPLC-MS was recorded on an inductively coupled plasma mass spectrometer XSeries II ICP-MS (Thermo Scientific Inc., Waltham, MA, USA). Melting points were determined using the melting point (temperature) apparatus Stuart SMP20 (Cole-Palmer, Stone, Staffordshire, UK).

For a qualitative analysis of reaction mixture compositions, aluminum TLC plates with silica gel (0.015–0.040 mm) with a fluorescent indicator F254 (20 × 20 cm^2^) (Merck Millipore, Darmstadt, Germany) were used. For preparative chromatographic separation of the substance mixtures, “Kieselgel 60” silica gel (0.015–0.040 mm, Merck Millipore, Darmstadt, Germany) was used.

### 2.4. Cell Culture

All cell lines were maintained in a CO_2_ incubator at 37 °C, 95% humidity, and 5% CO_2_. The composition of the culture medium for the cells was as follows: non-tumorigenic human embryonic kindey cells HEK 293 (ATCC CRL-1573), metastatic human breast cancer cells MDA-MB-231 (ATCC HTB-26), non-cancer immortalized human fibroblast cells Bj-5ta (ATCC CRL-4001), and human melanoma cells A-375 (ATCC CRL-1619): DMEM, 4 mM L-Gln, 10% fetal bovine serum (FBS), human glioblastoma cells U-87 MG (ATCC HTB-14): MEM, 2 mM L-Gln, 1% non-essential amino acids, 1 mM pyruvate, and 10% FBS; and human neuroblastoma cells SH-SY5Y (ATCC CRL-2266): 1:1 MEM: F12, 10% FBS, 2 mM L-Gln, 0.5 mM sodium pyruvate, and 0.5% non-essential amino acids. The cells were routinely checked for mycoplasma contamination using RT-PCR. All cell media contained 100 U/mL of penicillin, 100 µg/mL of streptomycin, and 2.5 µg/mL of amphotericin B. The cells were passaged using Trypsin–EDTA solution (PanEco, Moscow, Russia); the continuous passaging time did not exceed 40 passages. The cell lines were obtained from the ATCC.

Mycoplasma contamination was controlled using a Mycoplasma Detection Kit (Jena Bioscience, Jena, Germany).

### 2.5. Cytotoxicity and Proliferation Stimulation

For the analysis of cell death induction, the cells were plated in 96-well plates at a density of 1.5 × 10^4^ cells for the cytotoxicity assay and 8000 for the proliferation study per well and grown overnight. The dilutions of the test compounds prepared in DMSO and dissolved in the culture medium (without serum starvation) were added to the cells in triplicate for each concentration (100 µL of the fresh medium with the substance to 100 µL of the old medium in the well) and incubated for 24 h in the case of cytotoxicity and 72 h in the case of the proliferation stimulation. Of note, 24 h incubation time is generally enough for the manifestation of apoptosis-related and necrosis-related cell death, while detecting the effects on cell proliferation requires more time. The final DMSO concentration was 0.5%. Negative control cells (100% viability) were treated with 0.5% DMSO. Positive control cells (100% cell death) were treated with 3.6 μL of 50% Triton X-100 in ethanol per 200 μL of the cell culture medium. Separate controls were used without DMSO (no difference with the control 0.5% DMSO was found).

### 2.6. Cell Viability Assay

To evaluate cell death, an assay for the activity of the intracellular enzyme lactate dehydrogenase released into the medium from the dead cells was used [21]. To this end, a 75 µL aliquot of the culture medium from each well was transferred to a fresh 96-well plate. After that, 10 µL of the following reagents were added to each well: 36 mg/mL of lactate in phosphate-buffered saline, pH 7.2; 2 mg/mL of INT in the diaphorase buffer (see below); and 3 mg/mL of NAD^+^ mixed with 6 U/mL diaphorase in 0.03% bovine serum albumin and 1.2% sucrose in phosphate-buffered saline, pH 7.2. The reaction mixture was incubated for 20 min at room temperature, and the optical density of the solutions was determined at the wavelength of 490 nm using a Hidex Sense Beta Plus microplate reader (Hidex, Turku, Finland). The positive control was the cell culture treated with the solvent alone, and the negative control was treated with 0.9% Triton X-100.

### 2.7. Cell Death Assay

To evaluate cell death, the culture medium in the wells was replaced with a 0.2 mM resazurin solution in Earle’s solution with the addition of 1 g/l D-glucose and incubated for 1.5 h at 37 °C under cell culture conditions [22]. Thereafter, the fluorescence of the solution was determined at the excitation wavelength of 550 nm and the emission wavelength of 590 nm using a Hidex Sense Beta Plus microplate reader (Hidex, Turku, Finland). The positive control was the cell culture treated with the solvent alone, and the negative control was treated with 0.9% Triton X-100.

### 2.8. Molecular Docking

Ligand structures were obtained from the PubChem database (https://pubchem.ncbi.nlm.nih.gov/, accessed on 1 August 2024) or prepared manually using Avogadro 1.93.0 software and optimized using OpenBabel 3.0.0 software (http://openbabel.org/, accessed on 1 August 2024) [23], using the FFE force field with Fastest descent and dE ≤ 5 × 10^−6^ threshold. Protein structures were obtained from the PDB database (https://www.rcsb.org/, accessed on 1 August 2024) and optimized using the Chiron service (https://dokhlab.med.psu.edu/chiron/processManager.php, accessed on 1 August 2024) [24]. Molecular docking was performed using the AutoDock Vina 1.1.2 (http://vina.scripps.edu/, accessed on 1 August 2024). The grid center coordinates and sizes are represented in Table 1, and exhaustiveness was set to 8.

### 2.9. Statistics

All experiments were performed at least in triplicate. Statistical analysis was performed with GraphPad Prism 9.0 software using ANOVA with the Holm–Sidak or Tukey post-tests; *p* ≤ 0.05 was considered a statistically significant difference.

## 3. Results

### 3.1. Compound Synthesis

**Scheme 1:**
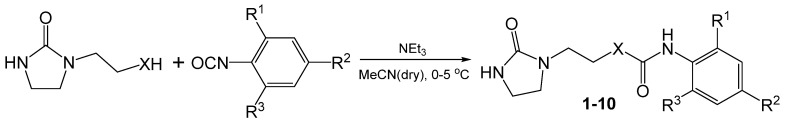
Chemical synthesis of EDU derivatives (**1**–**10**).

EDU derivatives **1**–**10** (Table 2 and Table 3) were obtained from 2-imidazolidinone with an aminoethyl or hydroxyethyl substituent through reaction with various aryl isocyanates in the presence of triethylamine in anhydrous acetonitrile, as described in [6,13] (Scheme 1). The synthesis procedure consisted of the reaction of key compounds with corresponding aryl isocyanates in the presence of triethylamine in anhydrous toluene (for aryl ureas) or in acetonitrile (for aryl carbamates). The yields for the compounds varied from 18 to 59%. This variation is typical for compounds with different substituents and depends on the purification methods required for each compound.

The structures of all synthesized compounds were confirmed using ^1^H NMR and mass-spectrometry analysis data. ^1^H NMR-spectra were recorded with a “Bruker DRX-400” spectrometer operating at 400.13 MHz frequency, using DMSO-d6 as the solvent and TMS as an internal standard. Chemical shifts were measured with 0.01 ppm accuracy, and coupling constants are reported in Hertz. HPLC-MS was recorded on an inductively coupled plasma mass spectrometer, XSeries II ICP-MS (Thermo Scientific Inc., USA). The melting points were determined by using the melting point (temperature) apparatus Stuart SMP20 (UK).

### 3.2. Influence on Cell Proliferation

First, we tested the synthesized compounds for their ability to induce cell death or decrease proliferation in a set of cancer cell lines. We used human cell lines of three major cancer types (glioblastoma U-87 MG, melanoma A-375, and metastatic breast cancer MDA-MB-231) and a neuroblastoma SH-SY5Y cell line in an acute 24-incubation setting. The cell lines were chosen to represent the major clinically significant cancer types. For the preliminary evaluation, the 24 h incubation time was chosen as it is typically enough for the compounds to modulate cell proliferation. However, in the case of the anti- or pro-proliferative action, a longer incubation period is typically required to reliably detect small changes in cell viability. Thus, in the case of substantial activity, additional experiments were carried out to evaluate the activity of the compounds after 72 h of incubation to obtain more details on the effects on cell proliferation. The compounds were assayed in the concentration range of 1–100 µM to account for the lowest potential load for the patient’s organism.

**Figure 2:**
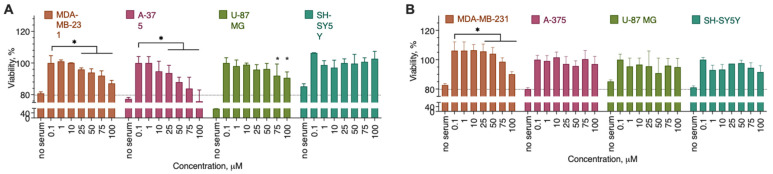
The anti-proliferative effect of the most active compounds, **6** (**A**) and **8** (**B**), on the MDA-MB-231, U-87MG, A-375, and SH-SY5Y cells during short-term incubation (24 h). Negative control cells (100% viability) were treated with 0.5% DMSO. Positive control cells (0% cell proliferation) were incubated in the cell culture medium without serum. Resazurin test data, amalgamated data of N = 3 experiments, mean ± standard error. *, a statistically significant difference from the control without the substance, *p* < 0.05 in ANOVA with the Tukey post-test.

**Figure 3:**
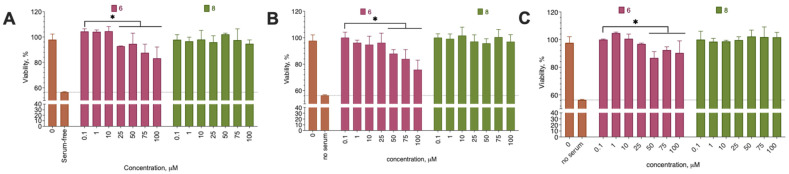
The anti-proliferative effect of the most active compound, **6**, on the MDA-MB-231 (**A**), A-375 (**B**), and U-87 MG (**C**) cells during long-term incubation (72 h). Negative control cells (100% viability) were treated with 0.5% DMSO. Positive control cells (0% cell proliferation) were incubated in the cell culture medium without serum. Resazurin test data, amalgamated data of N = 3 experiments, mean ± standard error. *, a statistically significant difference from the control without the substance, *p* < 0.05 in ANOVA with the Tukey post-test.

The only active compounds were compounds **6** and **8** (Table 4, Supplementary Figure S1). Compound **6** had an anti-proliferative effect on the cell lines MDA-MB-231, U-87 MG, and A-375, decreasing the cell viability by about 20% (up to 100% proliferation decrease compared to the non-proliferating control in serum-free medium) at 100 µM. Compound **8** had an anti-proliferative effect on MDA-MB-231, decreasing the cell viability by about 20% at 100 µM (Figure 2). Under prolonged incubation, compound **6** reduced proliferation by the same percentage (Figure 3).

Given that structurally similar compounds [5,19,22,25] are sometimes able to act as anti-oxidants, we tested the synthesized compounds in two anti-oxidant models (protection of the SH-SY5Y cell line against the H_2_O_2_ and CoCl_2_ cytotoxicity) in a 24 h incubation period. Neither substance was active (Supplement Figures S2 and S3).

Based on the highest observed activity, compound **6** was chosen for further evaluation as the most active one.

To validate whether compound **6** induces cell death in parallel to the anti-proliferative action, we performed a lactate dehydrogenase (LDH) test for cell death on the cell lines MDA-MB-231, A-375, and U-87 MG.

**Figure 4:**
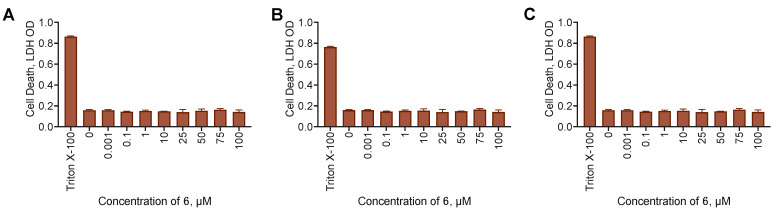
Cell death induction by compound **6** for the MDA-MB-231 (**A**), A-375 (**B**), and U-87 MG (**C**) cell line. Negative control cells (100% viability) were treated with 0.5% DMSO. Positive control cells (100% cell death) were treated with 3.6 μL of 50% Triton X-100 in ethanol per 200 μL of the cell culture medium. LDH test data, 72 h incubation time. Amalgamated data of *N* = 3 experiments.

Compound **6** treatment did not produce any LDH activity in the cell medium, indicating that the effects of the compound were purely anti-proliferative (Figure 4).

### 3.3. Selectivity of Active Compound ***6***

**Figure 5:**
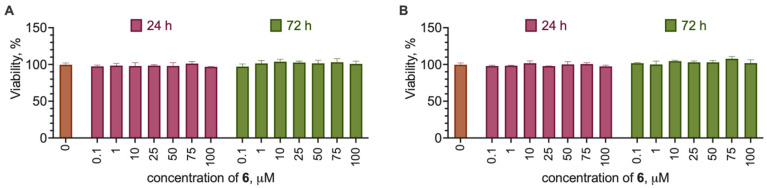
The effect of compound **6** on the viability of the human immortalized fibroblast Bj-5ta cell line (**A**) and HEK 293 cell line (**B**). Negative control cells (100% viability) were treated with 0.5% DMSO. Positive control cells (100% cell death) were treated with 3.6 μL of 50% Triton X-100 in ethanol per 200 μL of the cell culture medium, 24 and 72 h incubation time. Resazurin test data. Mean ± standard error (*N* = 3 experiments).

To investigate the selectivity of compound **6**, we used normal immortalized human fibroblast cell line Bj-5ta and the non-tumorigenic cell line HEK 293 in the same experimental setting as in the cytotoxicity studies. Compound **6** did not influence the proliferation of this cell line (Figure 5) either at 24 h or at 72 h incubation time.

### 3.4. Molecular Docking

To evaluate the possible mechanism of the anti-proliferative and cytoprotective action of the synthesized compounds, we performed a series of molecular docking experiments. We hypothesized that the active compounds and their molecular prototypes cytokinins could share at least some of the receptors and used these proteins as the targets.

The core proliferation-related target of cytokinins, the molecular prototypes of the synthesized compounds, is the adenosine A2 receptor (A2AR). We performed molecular docking of compound **6** to three variants of this receptor: two with blockers theophylline (5mzj [25]) and caffeine (5mzp [25]) and one with activator adenosine (2ydo [26]). We also compared the affinity scores with caffeine and adenosine. The molecular docking procedure is a computational experiment, which aims to estimate the affinity of a compound for a given protein molecule. In the variant used in this paper, we suggested that the binding site of the molecule is the receptor’s active site and limited the possible interaction areas to this protein part. To make the results more representative, the resultant affinities were compared to those of the known protein agonists and blockers. In addition, the crystal structures of the target receptor bound with both agonists and antagonists were used in a hypothesis, that the protein conformations in the activated and inhibited state could differ, and the different affinity of a compound to one of these states could indicate the compound’s activity mode.

**Figure 6:**
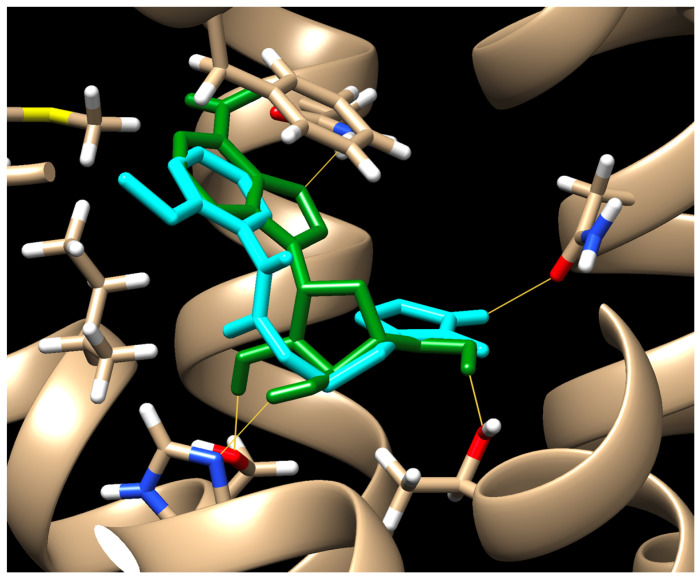
Molecular docking results for adenosine (green) and compound **6** (cyan) in the active site of the activated A2AR receptor. Red, oxygen; white, hydrogen; blue, nitrogen. AutoDock Vina data; hydrogen bonds are shown in yellow.

For all A2AR receptor variants, compound **6** demonstrated higher affinity scores that were better than any of those of adenosine and caffeine (Table 5). However, in the activated conformation, compound **6** formed only one hydrogen bond compared to adenosine, which formed four (Figure 6). The inability of the compound to form additional hydrogen bonds may be interpreted as follows: the compound binds to the receptor with the same affinity as the natural agonist. However, it does not form all of the possible hydrogen bonds and thus does not induce the same conformational changes of the protein as the agonist does.

### 3.5. Combined Activity with Doxorubicin

Anti-proliferative compounds can often enhance the activity of cytotoxic drugs. Such interactions are known, e.g., for curcumin and 5-fluorouracil, resveratrol, and cisplatin, as well as for various other natural compounds [27]. Given that compound **6**’s action was anti-proliferative, we expected that it could enhance the cytotoxic effects of the standard cytotoxic drug doxorubicin and the anti-glioblastoma drug temozolomide. To test this hypothesis, we evaluated the influence on cell viability during 72 h long incubation of various concentrations of doxorubicin with 90 and 100 µM of compound **6** in the breast cancer MDA-MB-231 and U-87 MG cell lines. As the action of temozolomide in our experimental setting was mostly anti-proliferative, we did not run an LDH test for its combinations with compound **6**.

**Figure 7:**
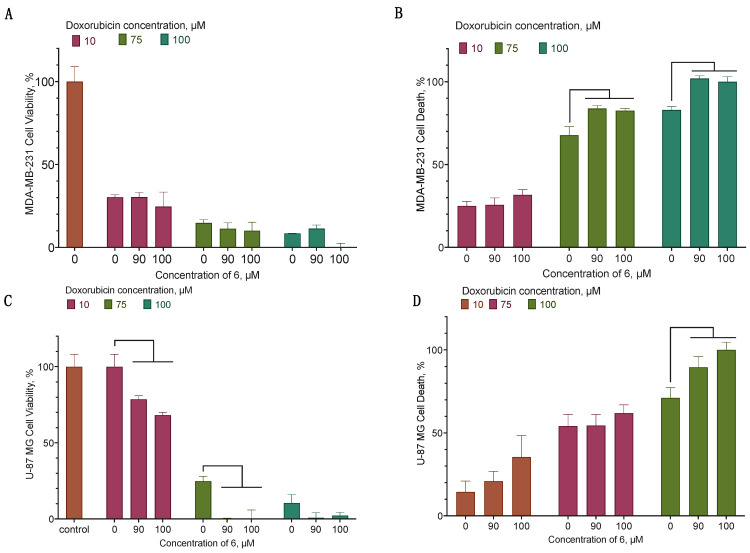
The effect of compound **6** on doxorubicin activity for the MDA-MB-231 (**A**,**B**) and U-87 MG (**C**,**D**) cell lines. Negative control cells (100% viability) were treated with 0.5% DMSO. Positive control cells (100% cell death) were treated with 3.6 μL of 50% Triton X-100 in ethanol per 200 μL of the cell culture medium. Note: 72 h incubation time, resazurin (**A**,**C**) and LDH (**B**,**D**) test data, mean ± standard error (N = 3 amalgamated experiments), *p* ≤ 0.05.

**Figure 8:**
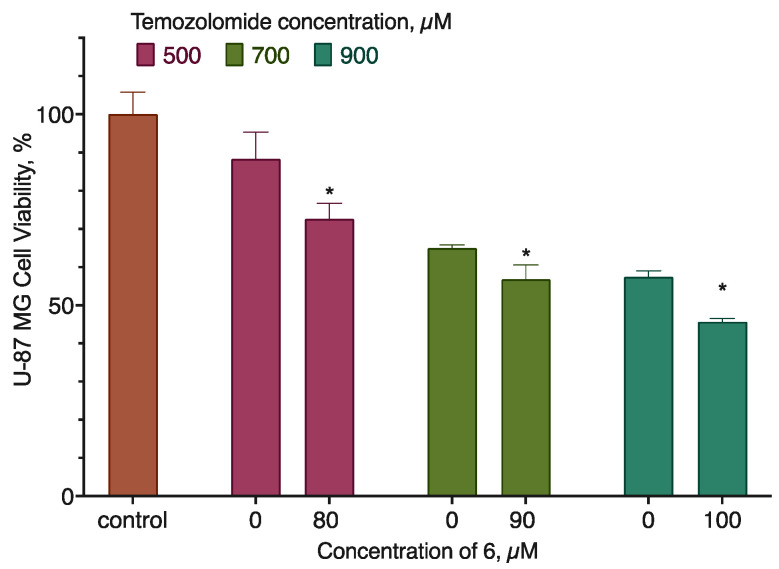
The effect of compound **6** on temozolomide activity for the U-87 MG cell line. Negative control cells (100% viability) were treated with 0.5% DMSO. Positive control cells (100% cell death) were treated with 3.6 μL of 50% Triton X-100 in ethanol per 200 μL of the cell culture medium. Note: 72 h incubation time, resazurin test data, mean ± standard error (N = 3 amalgamated experiments) * Statistically significant difference from the control without the substance in the ANOVA with the Dunnett’s post-test, *p* ≤ 0.05.

Combined with doxorubicin, compound **6** enhanced its cytotoxicity at higher concentrations by 20–30% in both cell lines (Figure 7). A similar activity enhancement was observed for the temozolomide combinations in U-87 MG (Figure 8).

## 4. Discussion

In this paper, we report the development of cytokinin analogs with anti-proliferative activity for breast cancer, melanoma, and glioblastoma cell lines. Based on our previous data [6,12,13], we hypothesized in this work that the modification of the ethylenediurea moiety with alkyl and oxygen-containing groups could be a way to enhance the molecule’s anti-proliferative properties.

To synthesize the designated derivatives, we used known methods with a yield in the range of 15 to 55%, which is typical for this type of compound.

The synthesized compounds were evaluated for their ability to induce cell death in a set of human cancer cell lines (glioblastoma U-87 MG, melanoma A-375, and metastatic breast cancer MDA-MB-231) chosen based on the clinical significance of the corresponding tumors and on the neuroblastoma SH-SY5Y cell line.

Most of the compounds displayed no activity, but the amino-linked compounds with C_2_H_5_ and OCH_3_ substituents were able to lower cell viability by 20%, making it close to the state of non-dividing cells cultivated without serum.

In general, the following trends in substance activity could be observed:

- Larger substituents (e.g., C_2_H_5_) and O linkers tended to result in lower viability (higher potency), particularly in breast cancer and melanoma.
- Smaller substituents (e.g., CH_3_) and NH linkers generally resulted in higher viability (lower potency).
- Electron-withdrawing groups (e.g., OCH_3_) may enhance activity compared to electron-donating groups (e.g., CH_3_).

In addition, C_2_H_5_ at R1 with an O linker was more effective against breast cancer and melanoma, while OCH_3_ at R2 with an NH linker showed promising activity against breast cancer and glioblastoma.

Neither of the compounds induced cell death, indicating the pure anti-proliferative action. The recalculated decrease in cell proliferation was up to 70–90%. This was substantially better than the activity of other similar molecules studied by us previously [6,12,13], and differed in a way that the novel compounds did not induce cell death.

The action of compound **6**, chosen for further mechanistic evaluation, was purely anti-proliferative, and it only affected the proliferation of cancer cells, not inhibiting normal immortalized fibroblasts or non-tumorigenic HEK 293 cells. To obtain more insights into the molecular mechanism of action of the active compounds, we performed molecular docking studies with the most widely known cytokinin analog target adenosine A2 receptor [28,29]. Based on their receptor affinity scores, the leading compound, **6**, was similar to both A2AR inhibitors and activators, indicating a high possibility of interaction with this protein. Compared to the agonist adenosine, compound **6** formed only one of four hydrogen bonds instead of four and thus possibly did not trigger the receptor conformation change, being an antagonist. This is in line with the pro-proliferative or pro-survival signaling of the adenosine receptors and the anti-proliferative effects of their blockers [30,31]. Previously, we observed similar effects for other cytokinin analogs [6]. However, more research is required to validate this target.

The observed active concentration range of compound **6** was from 50 to 100 µM. This concentration is higher than some of the known cytostatic compounds, like tamoxifen, ruxolitinib, and letrozole [32,33,34], which are typically active in the range from 0.1 to 10 µM. However, in the glioblastoma models, such activity is a typical one [35,36] and could be used as a ground for further structural optimization.

Given its anti-proliferative properties, we hypothesized that the activity of compound **6** could be complementary to the standard anticancer drugs and tested the combined activity of this substance with doxorubicin and temozolomide. As expected, the drug combinations reduced the viability of the cell cultures to a greater extent (by 20–30% compared to the doxorubicin or temozolomide alone). Both temozolomide and doxorubicin are DNA-damaging agents [37,38], with the second being able to induce oxidative stress as well. This means that their activity is more pronounced for the dividing cells and explains the limited enhancement by the developed compound. It could be suggested that the proposed cytokinin analog could be a better complement to another cytostatic drug or an immunomodulating agent.

The observed low non-specific toxicity combined with a rather simple synthesis procedure makes the proposed compounds an interesting target for further structural optimization and testing in combination with the aforementioned drugs.

## 5. Conclusions

This research aimed to test the hypothesis on the ability of alkyl and oxygen-containing substituents to enhance the anti-proliferative activity of chemical cytokinins of the ethylenediurea family. Using several human cancer cell lines representing the major cancer types (breast cancer, glioblastoma, and melanoma), we have shown that the introduction of a longer-chain (ethyl) substituent in the aromatic ring of the EDU molecule leads to a compound with pronounced anti-proliferative activity (up to 100% proliferation inhibition) and selectivity towards cancer. The observed activity was able to enhance the efficiency of the standard anticancer drugs temozolomide and doxorubicin by 10–20% and was realized via the potential inhibition of the adenosine receptor A2AR. While the results are promising, the synthesized compounds have a notable drawback: their active concentration is relatively high (in the range of 50–100 µM). This may limit their immediate clinical applicability, as achieving such concentrations in vivo could pose challenges related to toxicity, bioavailability, and pharmacokinetics.

The observed anti-proliferative activity of the ethyl-substituted EDU compounds against glioblastoma cells is particularly encouraging. These compounds could serve as a foundation for developing supporting or adjunct therapies to enhance the efficacy of existing treatments like temozolomide, which is the current standard of care but is often limited by resistance and side effects. The ability of these compounds to inhibit A2AR, a receptor implicated in immune evasion and tumor progression, further underscores their potential to overcome therapeutic resistance.

The enhancement of temozolomide and doxorubicin efficacy by 10–20% is clinically significant, as even modest improvements in drug efficacy can translate into better patient outcomes. In breast cancer, doxorubicin is a cornerstone of treatment, but its use is often limited by cardiotoxicity and resistance [23]. The novel EDU compounds could allow for lower doses of doxorubicin to achieve the same therapeutic effect, thereby reducing side effects. In melanoma, where resistance to targeted therapies and immunotherapies is common [24], the addition of EDU-derived compounds could provide a new avenue for combination therapy.

## Figures and Tables

**Table 1:** A2AR grid parameters in the docking experiments.

Protein Variant	Center			Size		
	X	y	z	X	y	z
5mzj	−20.1525	7.2827	18.3689	9.86947	11.555	10.1538
2ydo	−30.0788	6.16796	−22.041	12.1031	18.9539	10.608
5mzp	−20.9002	8.17906	17.4545	11.2522	14.6355	11.9605

**Table 2:** The structure and yields of the synthesized compounds.

Compound	R_1_	R_2_	R_3_	X	Yield, %
**1**	CH_3_	-	CH_3_	NH	59
**2**	CH_3_	-	CH_3_	O	30
**3**	-	CH_3_	-	NH	31
**4**	-	CH_3_	-	O	36
**5**	C_2_H_5_	-	-	NH	62
**6**	C_2_H_5_	-	-	O	50
**7**	CH_3_	CH_3_	-	NH	58
**8**	-	OCH_3_	-	NH	35
**9**	-	OCH_3_	-	O	18
**10**	-	COOCH_3_	-	O	30

**Table 3:** The structural formulas of the synthesized compounds.

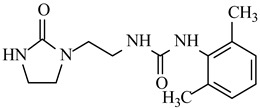 1	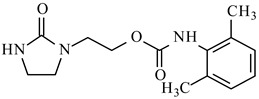 2
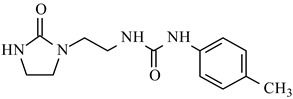 3	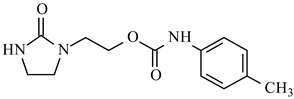 4
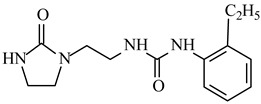 5	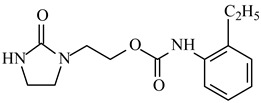 6
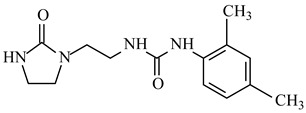 7	
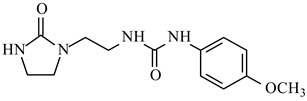 8	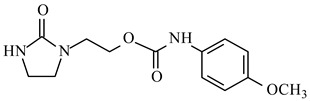 9
	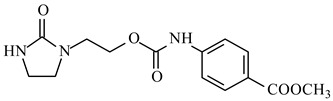 10

**Table 4:** The effect on cell proliferation of 100 µM of the synthesized compounds. Incubation time 24 h. Resazurin test data, amalgamated data of N = 3, mean ± standard error. *, a statistically significant difference from the control without the substance, *p* < 0.05 in ANOVA with the Tukey post-test.

Compound	MDA-MB-231	A-375	U-87 MG	SH-SY5Y
	Proliferation After 24 h Incubation with 100 µM, %			
No substance	100 ± 6.6	100 ± 7.7	100 ± 3.2	100 ± 5.8
**1**	76 ± 9.3 *	59.1 ± 6.6 *	65.1 ± 4.5 *	93.4 ± 6.9
**2**	84.9 ± 8.3	67.4 ± 6.7 *	70 ± 1 *	83.7 ± 4.3 *
**3**	76.1 ± 9.7 *	100 ± 6	85.9 ± 5.5 *	85.4 ± 5.6 *
**4**	61.9 ± 1.5 *	60.1 ± 3.7 *	55.4 ± 6.9 *	100 ± 10.1
**5**	66.4 ± 2 *	96.1 ± 5.9	100 ± 9.7	93 ± 6.7
**6**	26.9 ± 6.8 *	0 ± 4.1 *	44.2 ± 6.9 *	89.4 ± 6.2
**7**	100 ± 4.7	77 ± 10.3 *	62.7 ± 5.4 *	70.9 ± 7.4 *
**8**	37.9 ± 6.5 *	78.9 ± 4.6 *	77.1 ± 7.2 *	75.9 ± 5.2 *
**9**	65.7 ± 5.2 *	79.1 ± 4 *	60.1 ± 8.1 *	67.5 ± 10 *
**10**	73.9 ± 9.9 *	67.5 ± 7.2 *	54.1 ± 5.9 *	71.4 ± 5.8 *

**Table 5:** Affinity of the synthesized compounds for the adenosine A2 receptor crystal variants. AutoDock Vina data. A lower score means higher affinity.

	A2AR Inhibited (Theophylline)	A2AR Activated (Adenosine)	A2AR Inhibited (Caffeine)
	Affinity Score		
Adenosine (activator)	−6.197	−7.308	−6.538
Caffeine (blocker)	−6.183	−6.326	−6.054
Compound 6	−7.824	−7.898	−7.643

## Data Availability

The data presented in this study are available from the corresponding author upon request. The data are not publicly available due to legal issues.

## Supplementary Materials

The following are available online at https://www.mdpi.com/article/10.3390/biomedicines13020316/s1: Figure S1: The effect of the synthesized compounds on MDA-MB-231, U-87MG, A-375, and SH-SY5Y cells’ viability; Figure S2: The effect of the synthesized compounds on the cytotoxicity of CoCl_2_ for the SH-SY5Y cell line; Figure S3: The effect of the synthesized compounds on the cytotoxicity of H_2_O_2_ for the SH-SY5Y cell line; Figure S4: NMR, HPLC, and MS spectroscopy data for the synthesized compounds.
