# Supplementary material for: Anti-Proliferative Activity of Ethylenediurea Derivatives with Alkyl and Oxygen-Containing Groups as Substituents
Source: Biomedicines. 2025 Jan 29;13(2):316. doi: 10.3390/biomedicines13020316 (PMC11852481; doi:10.3390/biomedicines13020316)
Supplement: Supplementary file 1 [file biomedicines-13-00316-s001.zip › biomedicines-3410342-supplementary.pdf]

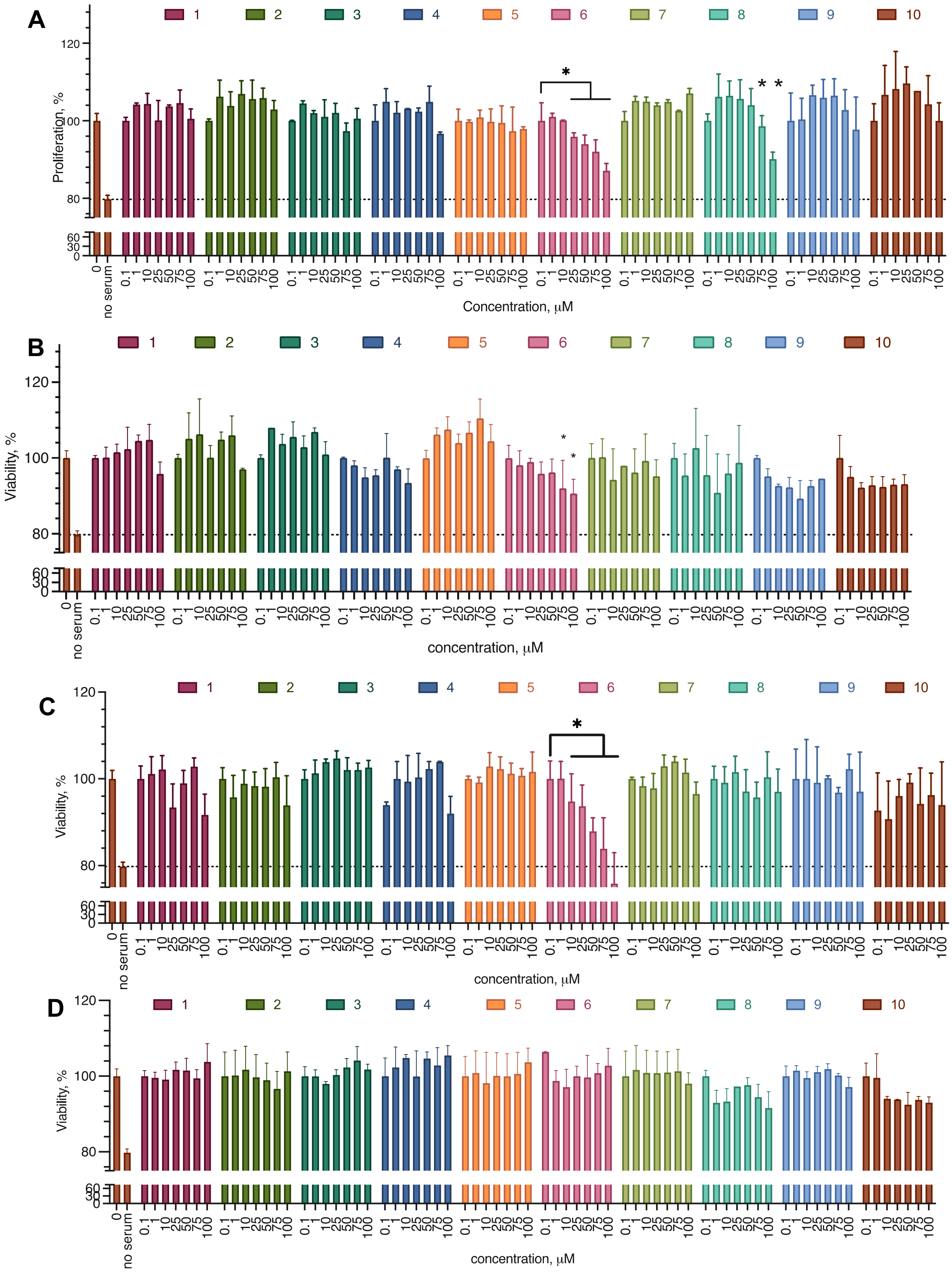

Figure S1. The effect of the synthesized compounds on the MDA-MB-231 (**A**), U-87MG (**B**), A-375 (**C**), and SH-SY5Y (**D**) cells viability. Incubation time 24 h. Resazurin test data, amalgamated data of N=3, mean±standard error. *, a statistically significant difference from control without substance, $p<0.05$ in ANOVA with the Tukey post-test

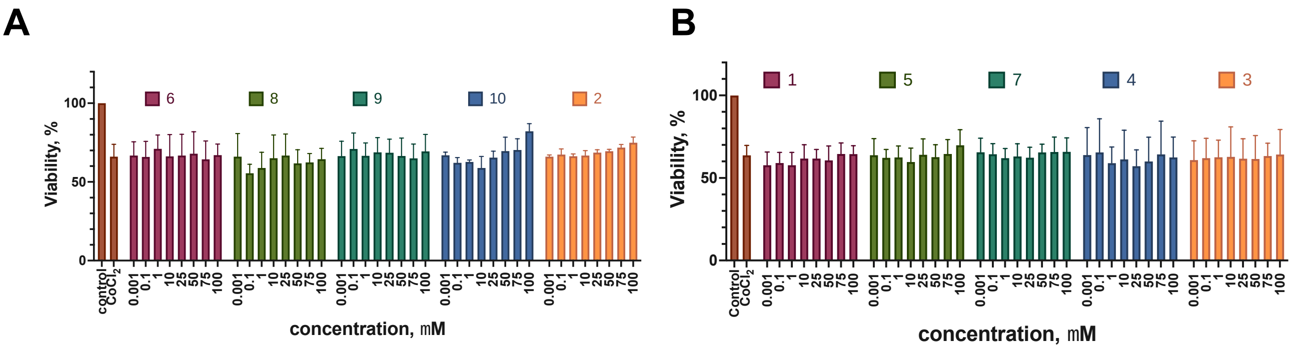

Figure S2. The effect of the synthesized compounds on the cytotoxicity of $CoCl_2$ for the SH-SY5Y cell line; 24 h incubation time, resazurin test data, amalgamated data of N=4 experiments, mean±standard error.

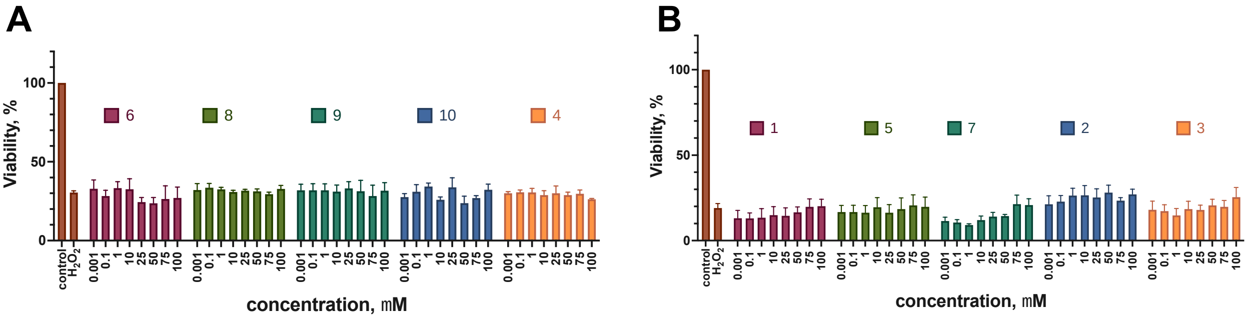

Figure S3. The effect of the synthesized compounds on the cytotoxicity of H_2O_2 for the SH-SY5Y cell line; 24 h incubation time, resazurin test data, amalgamated data of N=4 experiments, mean±standard error.

Figure S4.

2-(2-oxoimidazolidin-1-yl)ethyl-N-(2,6-dimethylphenyl) urea (1)

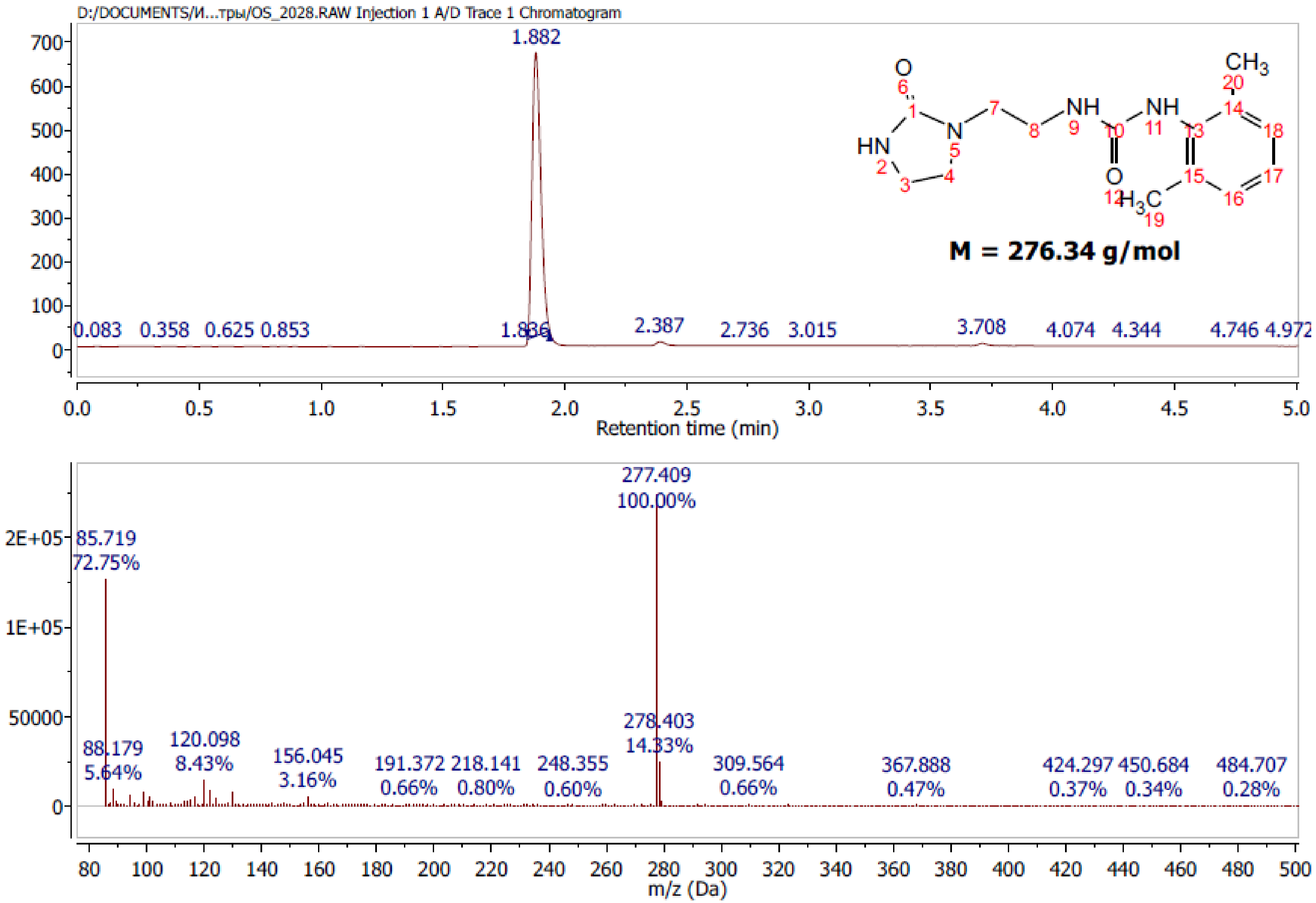

HPLC-MS spectrum of 2-(2-oxoimidazolidin-1-yl)ethyl-N-(2,5-dimethylphenyl) urea

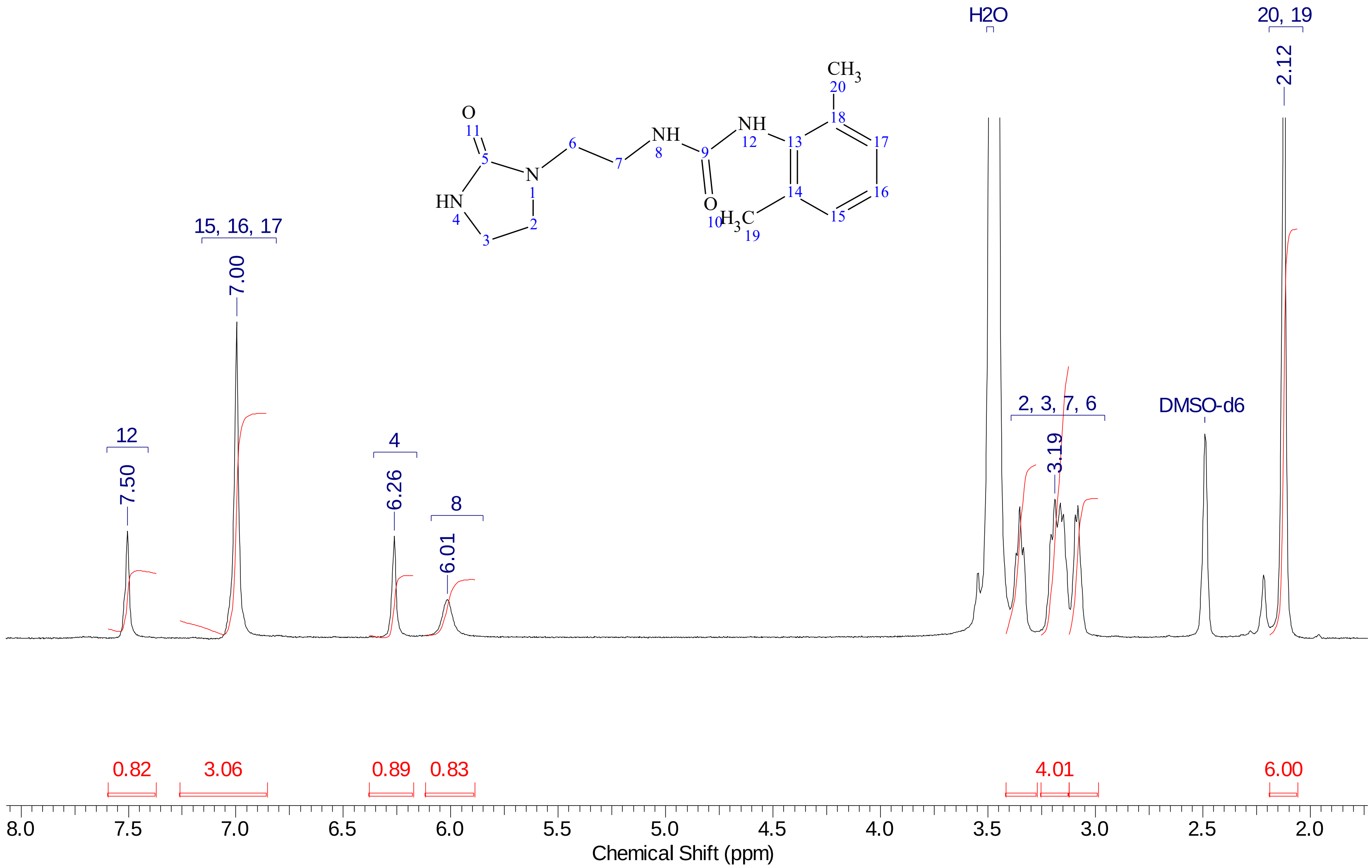

1H NMR spectrum of 2-(2-oxoimidazolidin-1-yl)ethyl-N-(2,5-dimethylphenyl) urea, DMSO-d6, 400 MHz

2-(2-oxoimidazolidin-1-yl)ethyl-N-(*p*-tolil) urea (3)

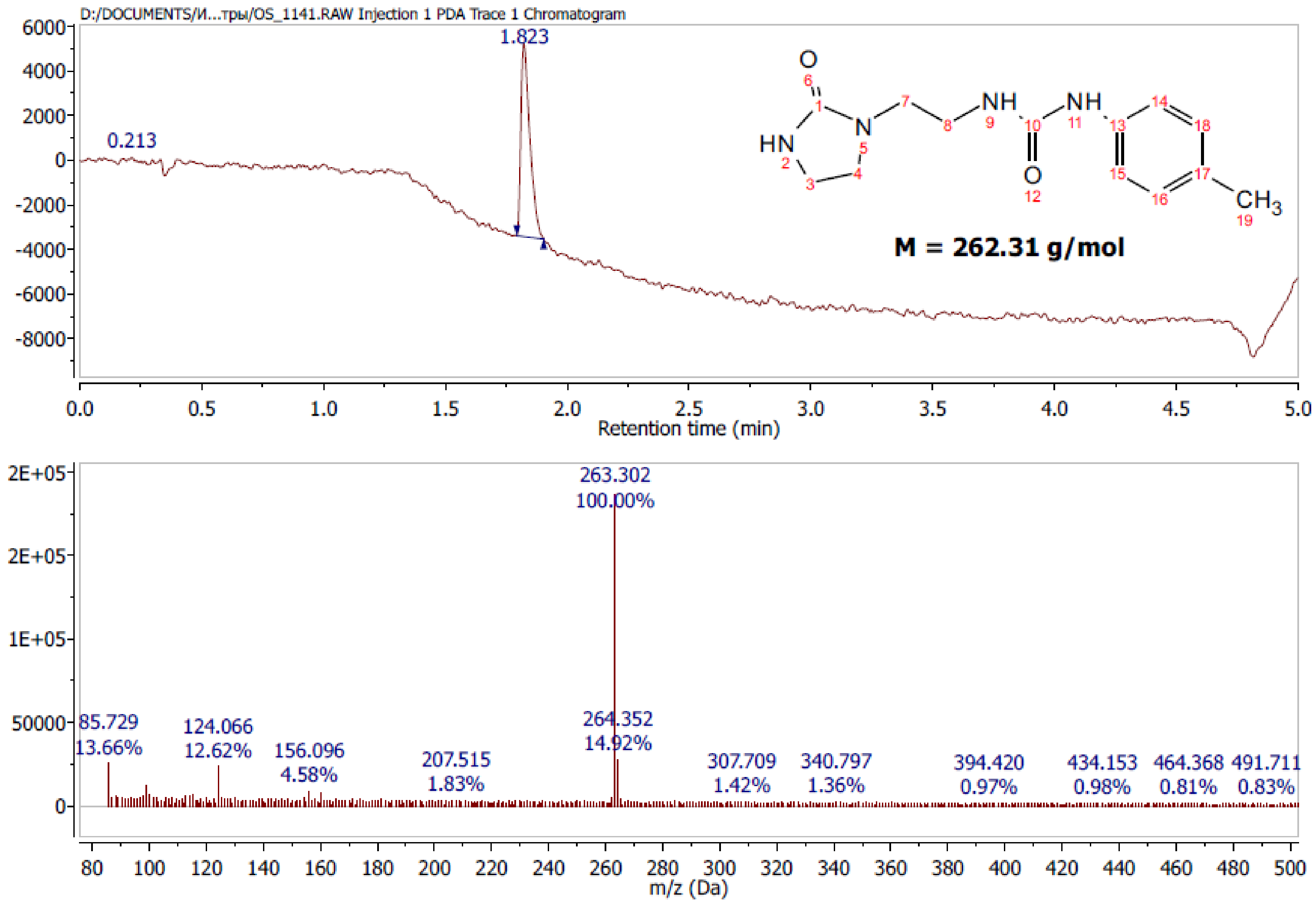

HPLC-MS spectrum of 2-(2-oxoimidazolidin-1-yl)ethyl-N-(*p*-tolil)urea

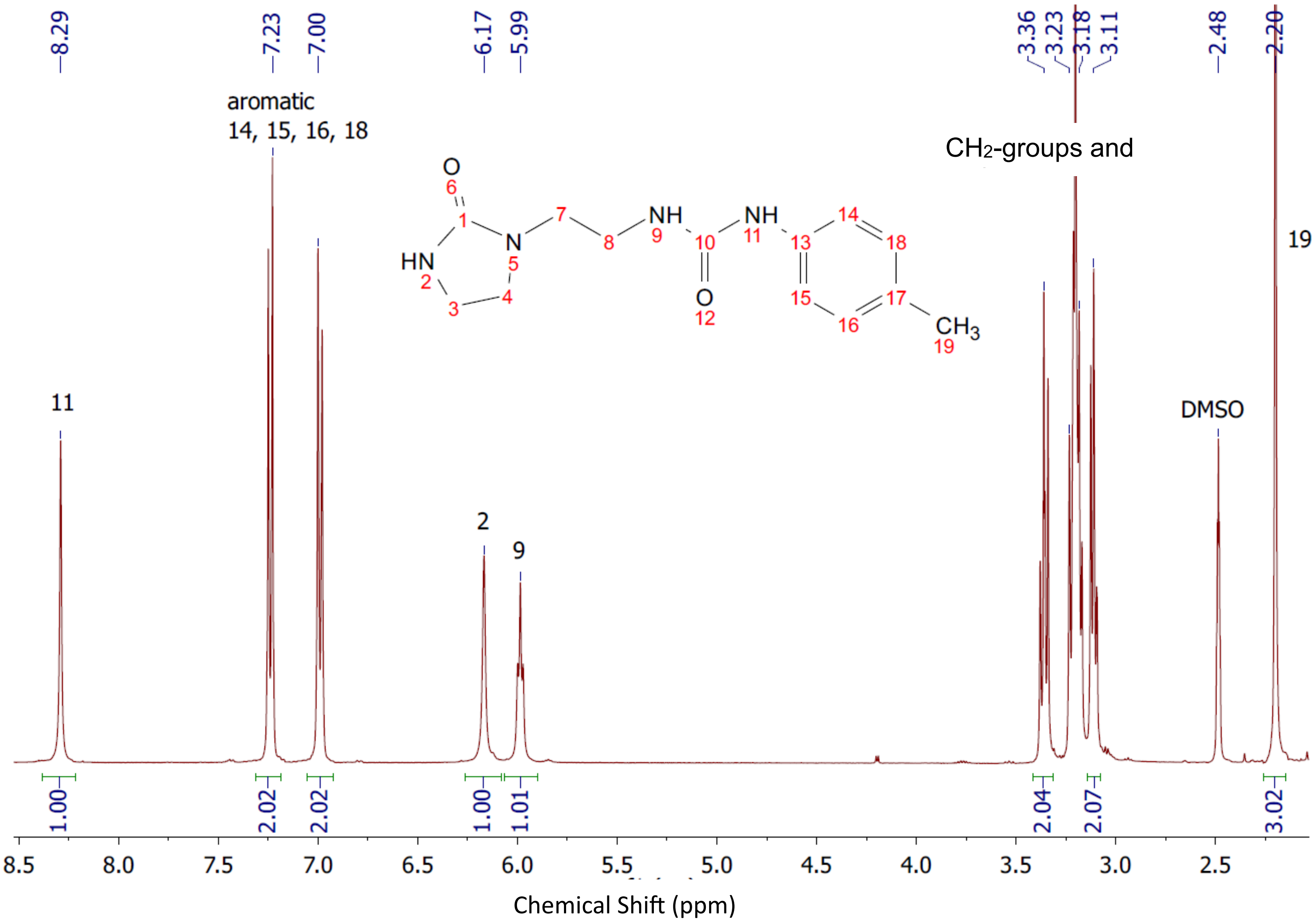

1H NMR spectrum of 2-(2-oxoimidazolidin-1-yl)ethyl-N-(p-tolil) urea, DMSO-d6, 400 MHz

2-(2-oxoimidazolidin-1-yl)ethyl-N-(2-ethylphenyl) urea (5)

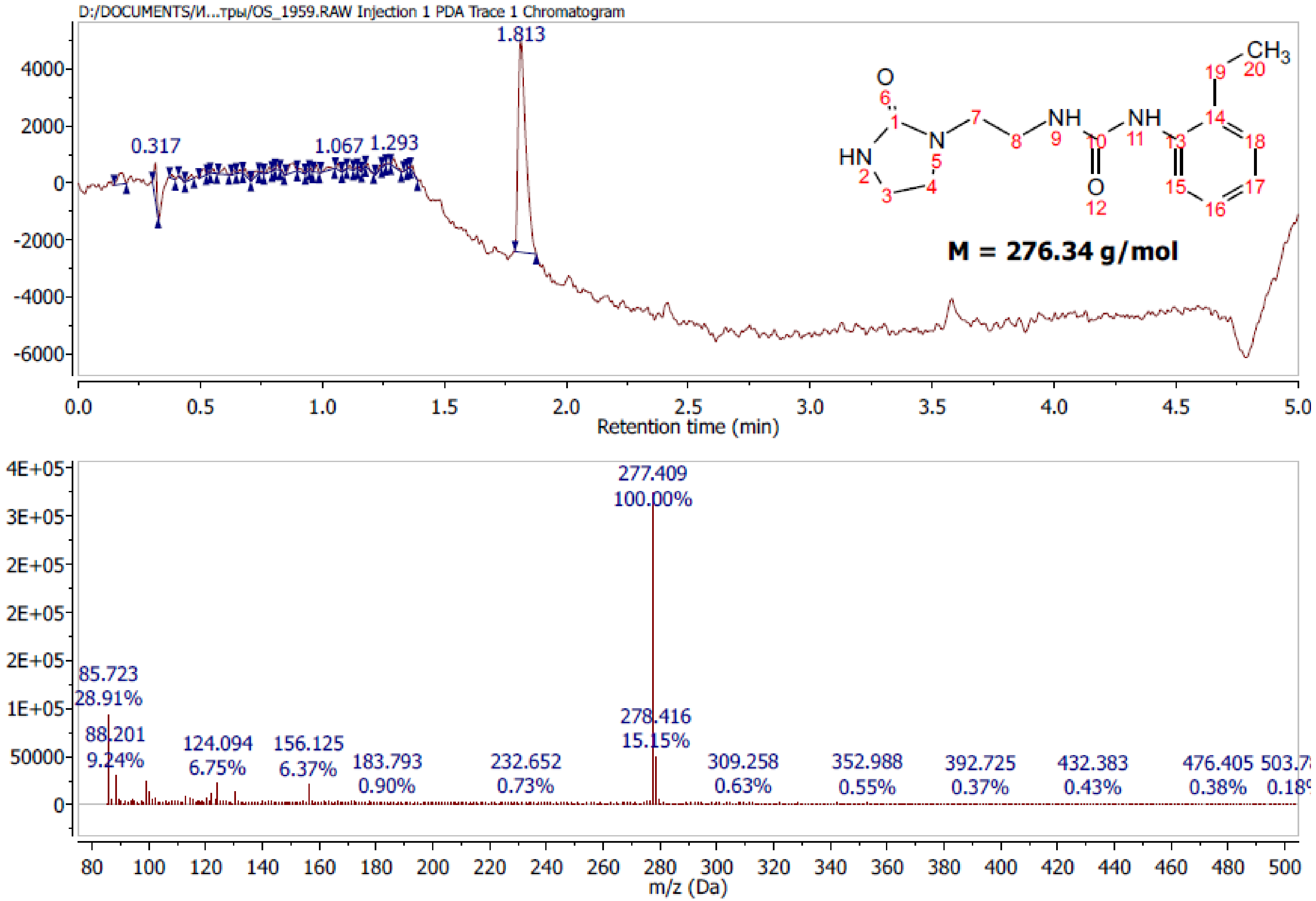

HPLC-MS spectrum of 2-(2-oxoimidazolidin-1-yl)ethyl-N-(2-ethylphenyl) urea

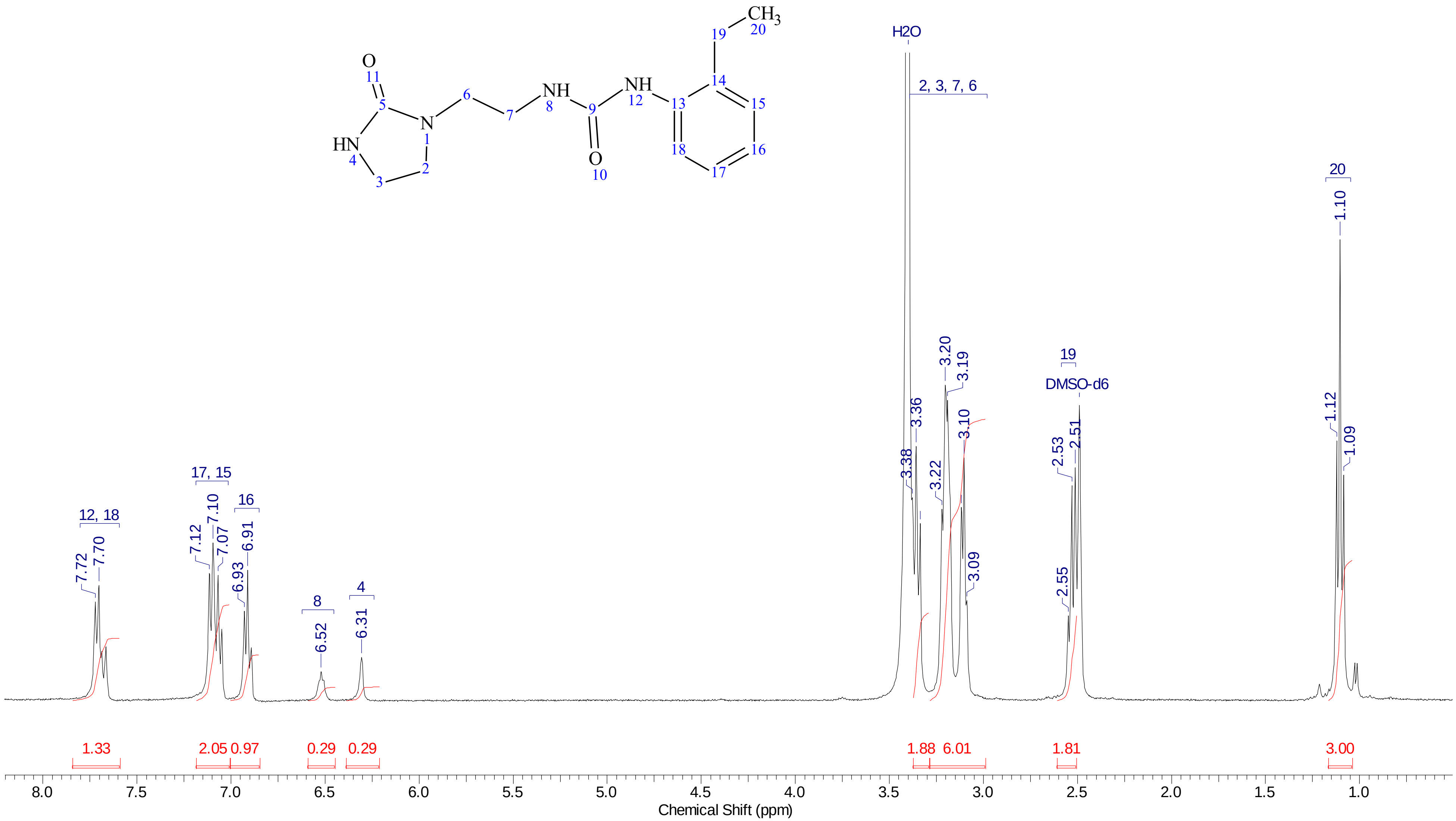

1H NMR spectrum of 2-(2-oxoimidazolidin-1-yl)ethyl-N-(2-ethylphenyl) urea, DMSO-d6, 400 MHz

2-(2-oxoimidazolidin-1-yl)ethyl-N-(2,4-dimethylphenyl) urea (7)

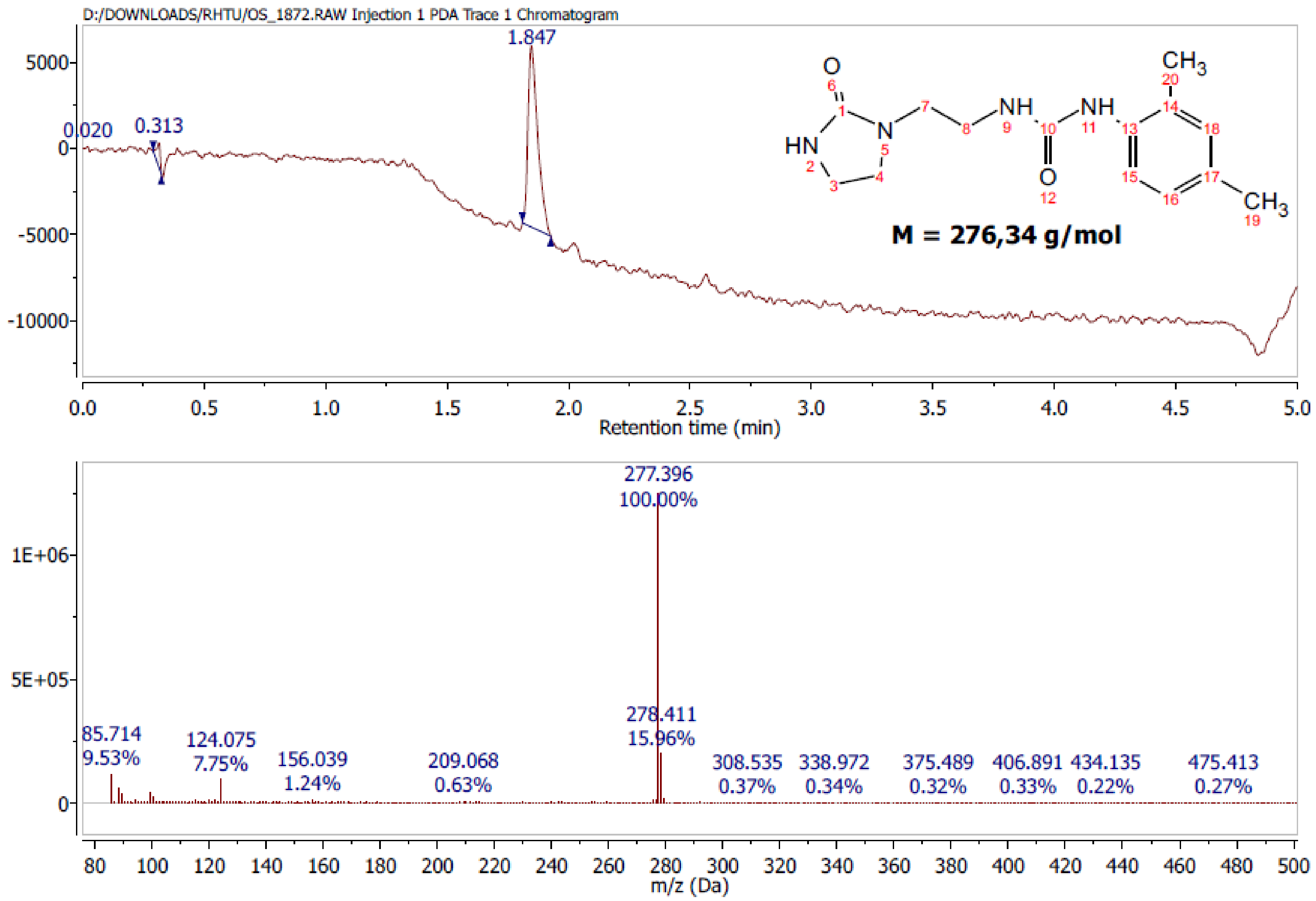

HPLC-MS spectrum of 2-(2-oxoimidazolidin-1-yl)ethyl-N-(2,4-dimethylphenyl) urea

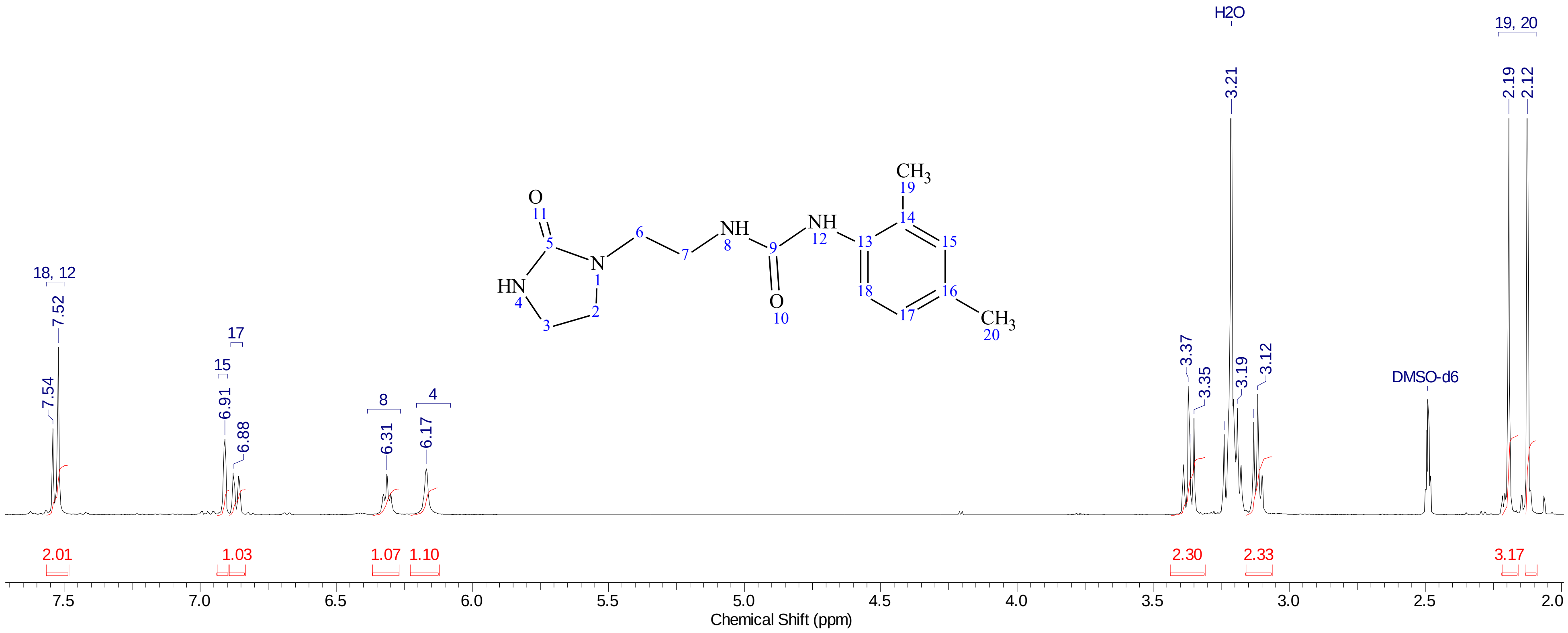

1H NMR spectrum of 2-(2-oxoimidazolidin-1-yl)ethyl-N-(2,4-dimethylphenyl) urea, DMSO-d6, 400 MHz

2-(2-oxoimidazolidin-1-yl)ethyl-N-(4-methoxyphenyl) urea (8)

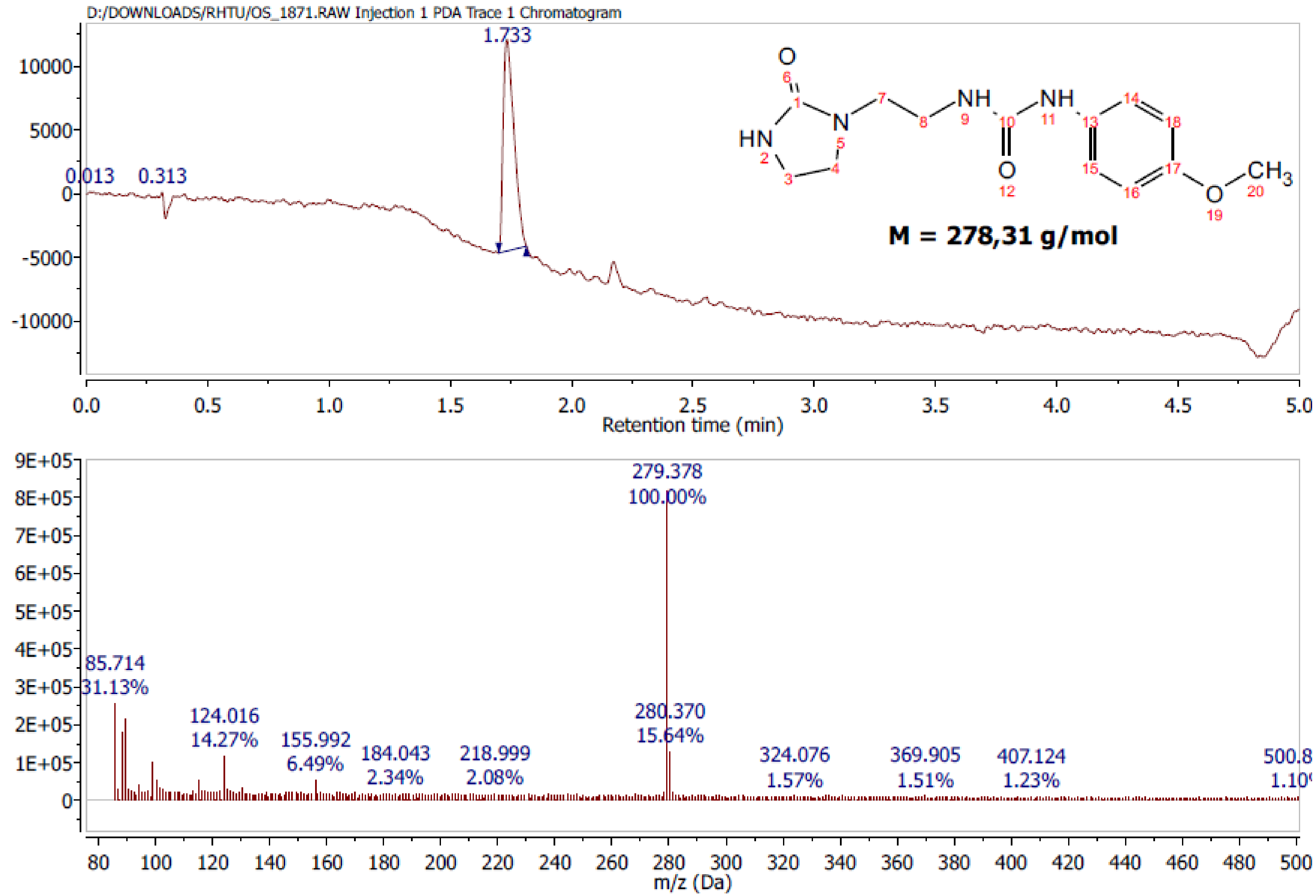

HPLC-MS spectrum of 2-(2-oxoimidazolidin-1-yl)ethyl-N-(4-methoxyphenyl) urea

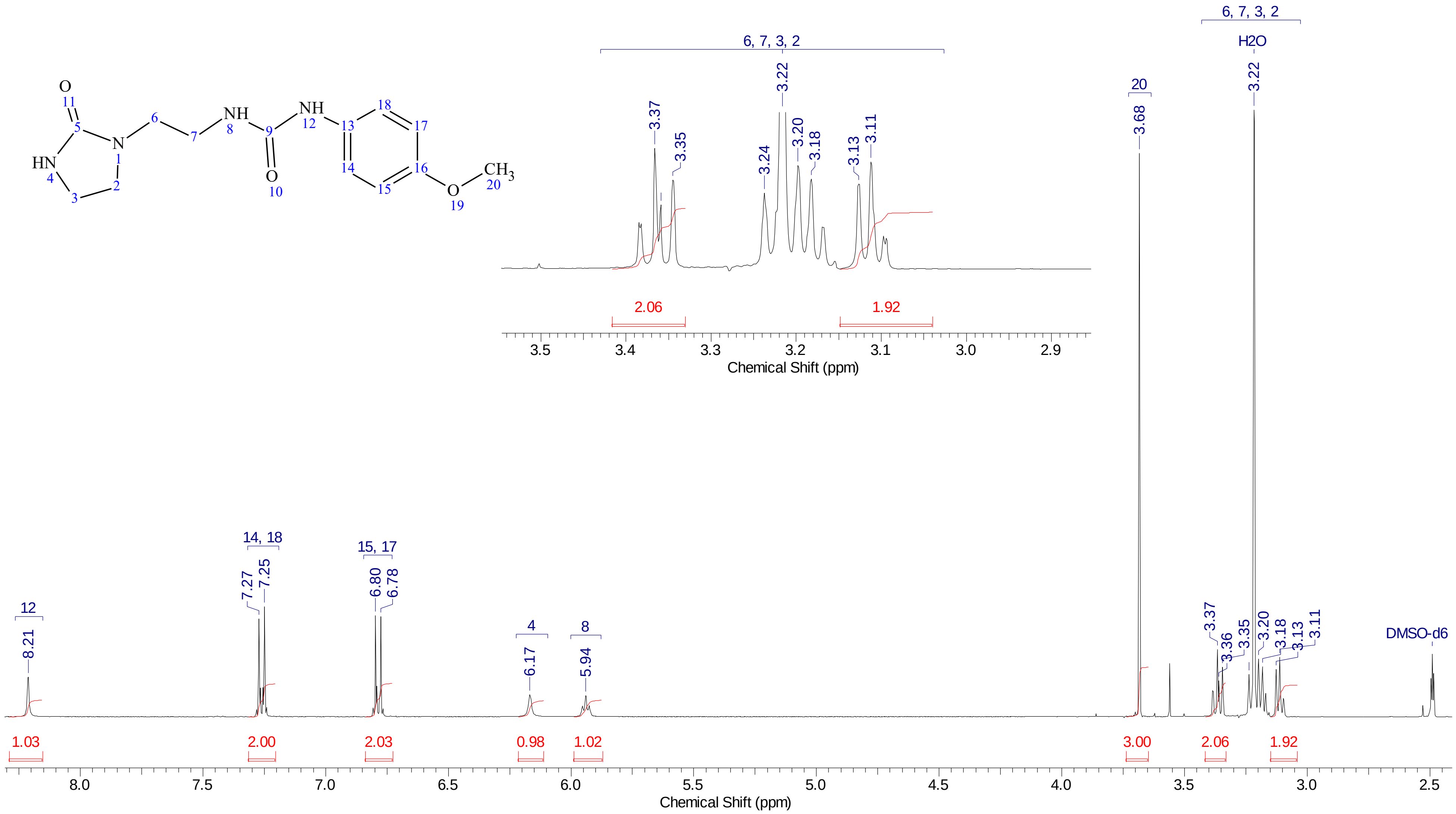

1H NMR spectrum of 2-(2-oxoimidazolidin-1-yl)ethyl-N-(4-methoxyphenyl) urea, DMSO-d6, 400 MHz

2-(2-oxoimidazolidin-1-yl)ethyl-N-(2,6-dimethylphenyl) carbamate (2)

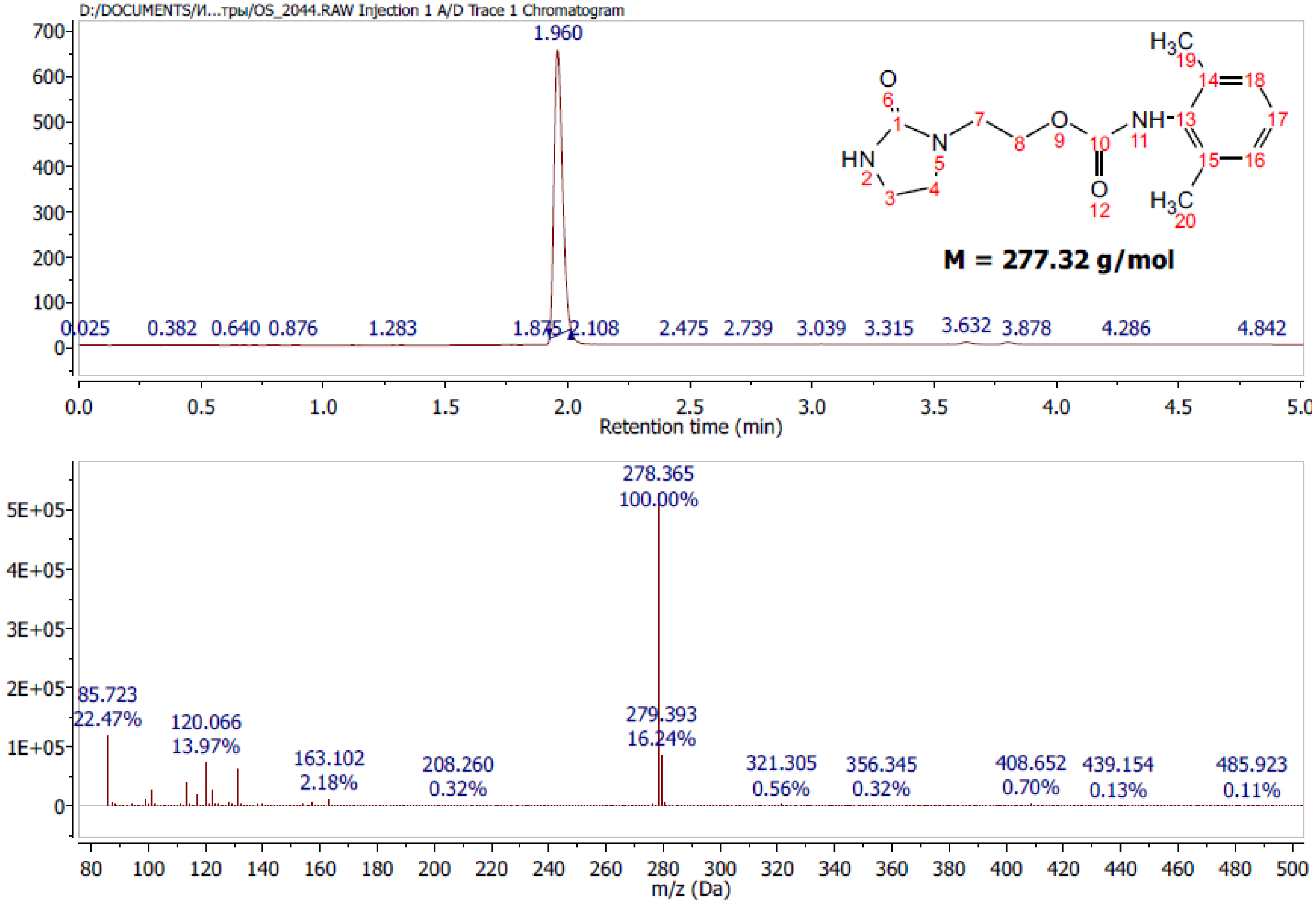

HPLC-MS spectrum of 2-(2-oxoimidazolidin-1-yl)ethyl-N-(2,6-dimethylphenyl) carbamate

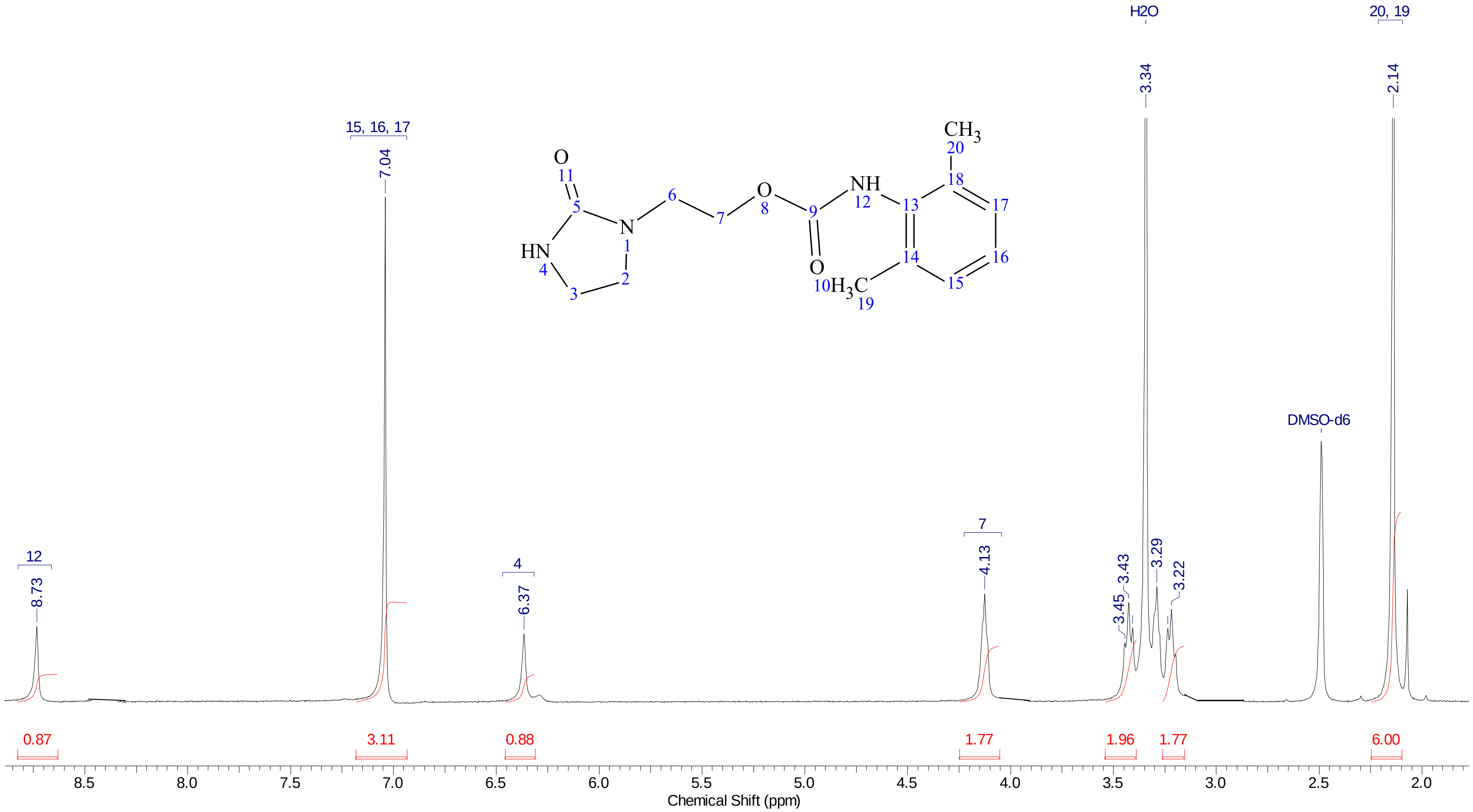

1H NMR spectrum of 2-(2-oxoimidazolidin-1-yl)ethyl-N-(2,6-dimethylphenyl) carbamate, DMSO-d6, 400 MHz

2-(2-oxoimidazolidin-1-yl)ethyl-N-(*p*-tolil) carbamate (4)

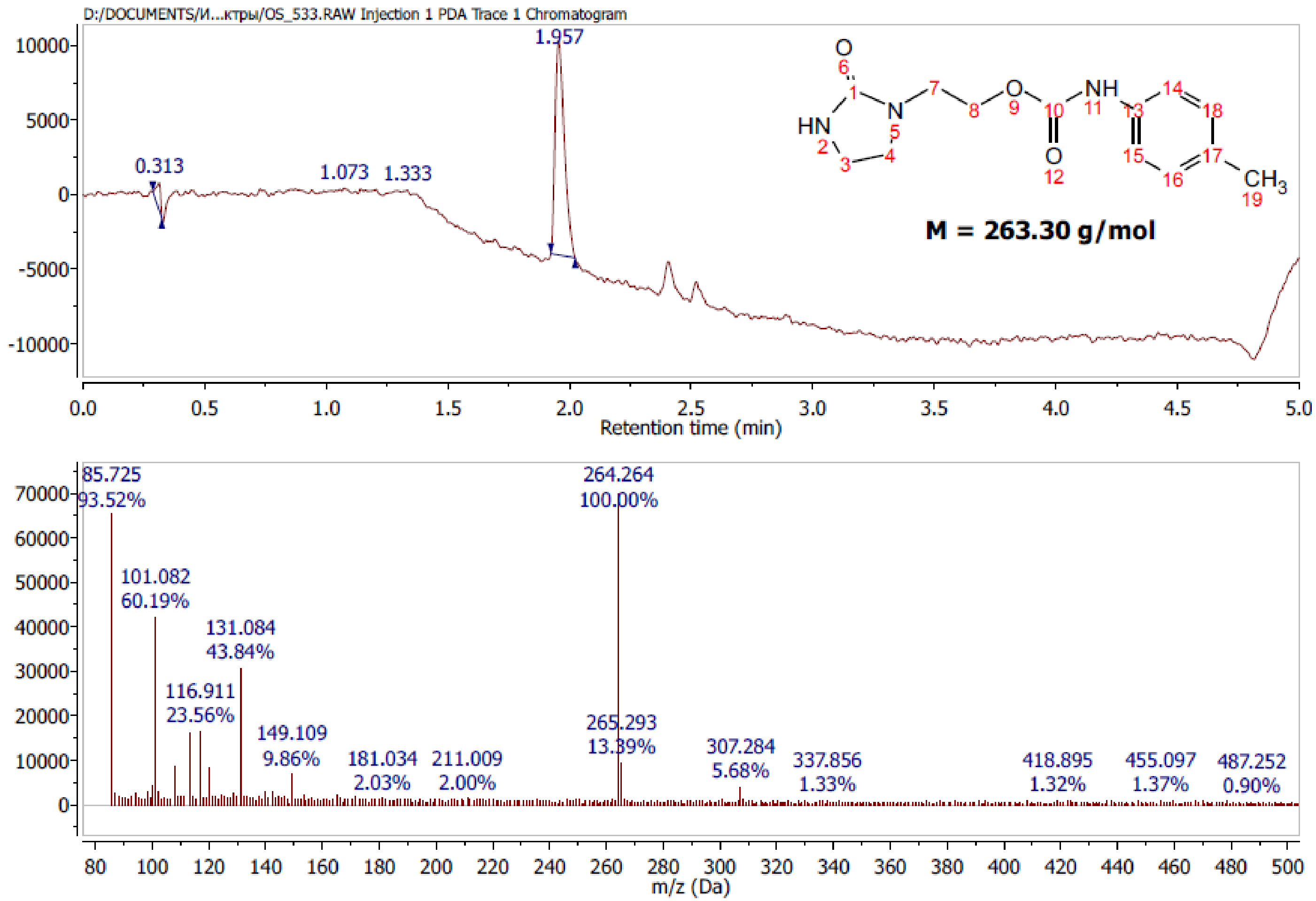

HPLC-MS spectrum of 2-(2-oxoimidazolidin-1-yl)ethyl-N-(p-tolil) carbamate

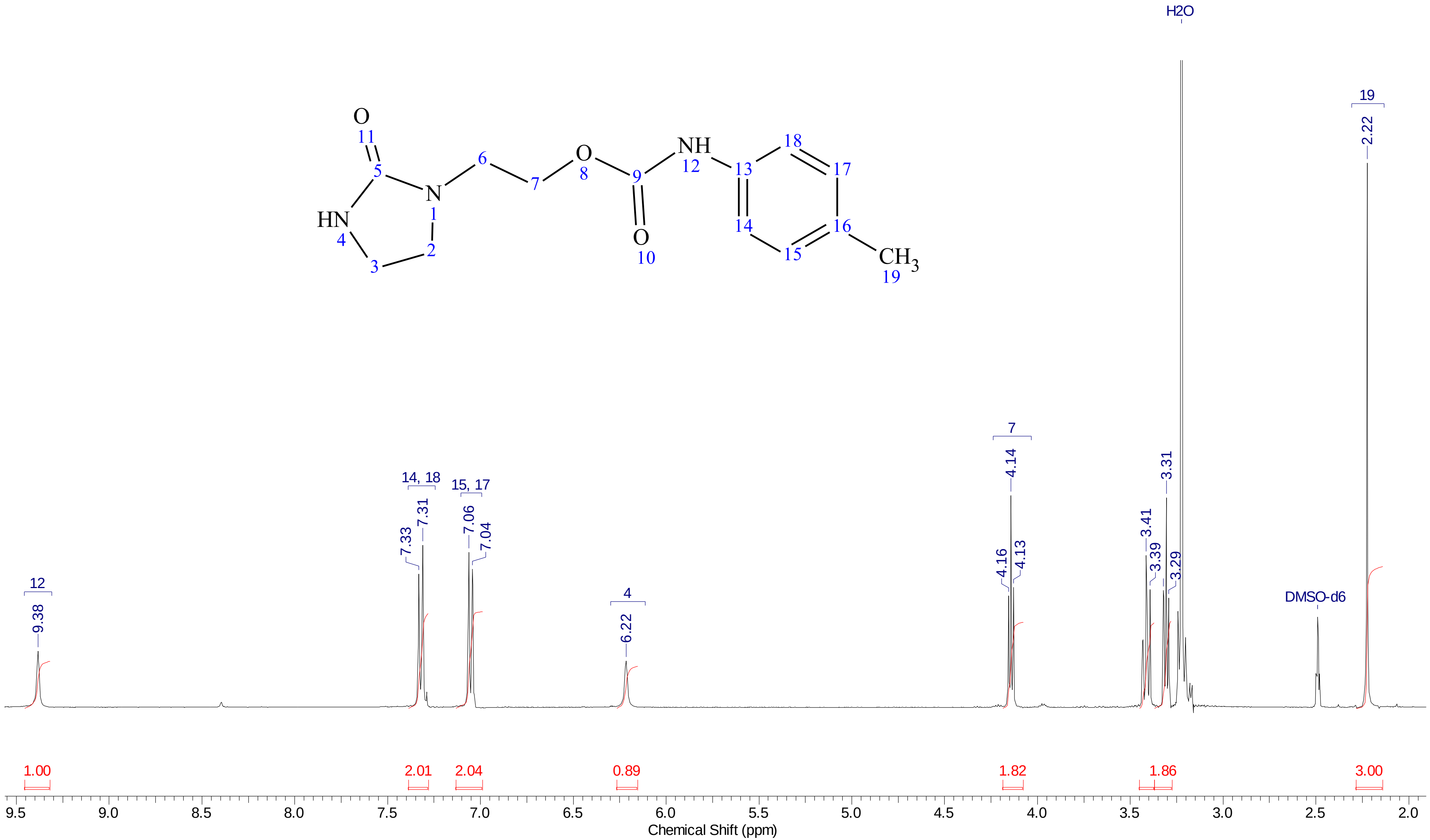

1H NMR spectrum of 2-(2-oxoimidazolidin-1-yl)ethyl-N-(p-tolil) carbamate, DMSO-d6, 400 MHz

2-(2-oxoimidazolidin-1-yl)ethyl-N-(2-ethylphenyl) carbamate (6)

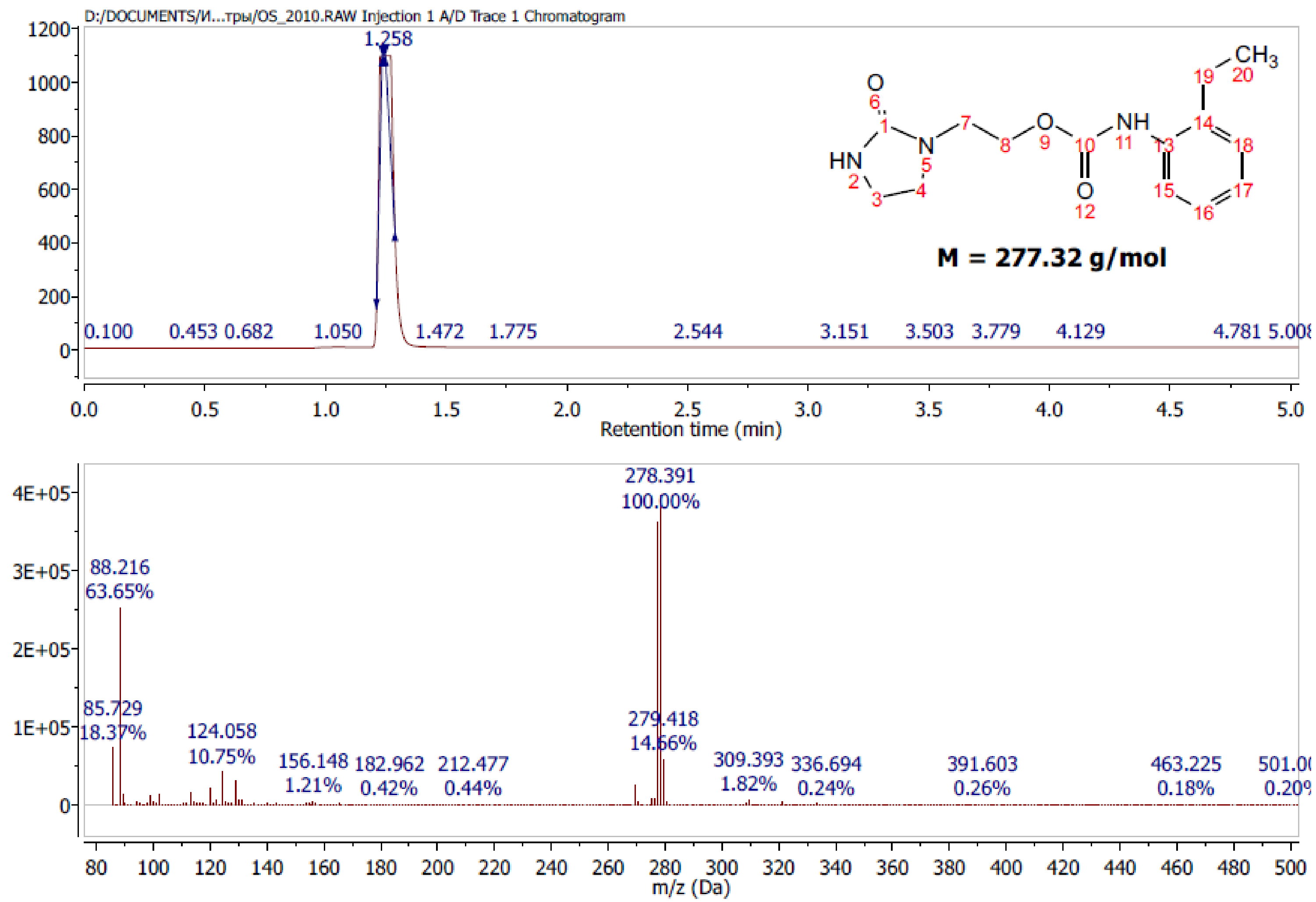

HPLC-MS spectrum of 2-(2-oxoimidazolidin-1-yl)ethyl-N-(2-ethylphenyl) carbamate

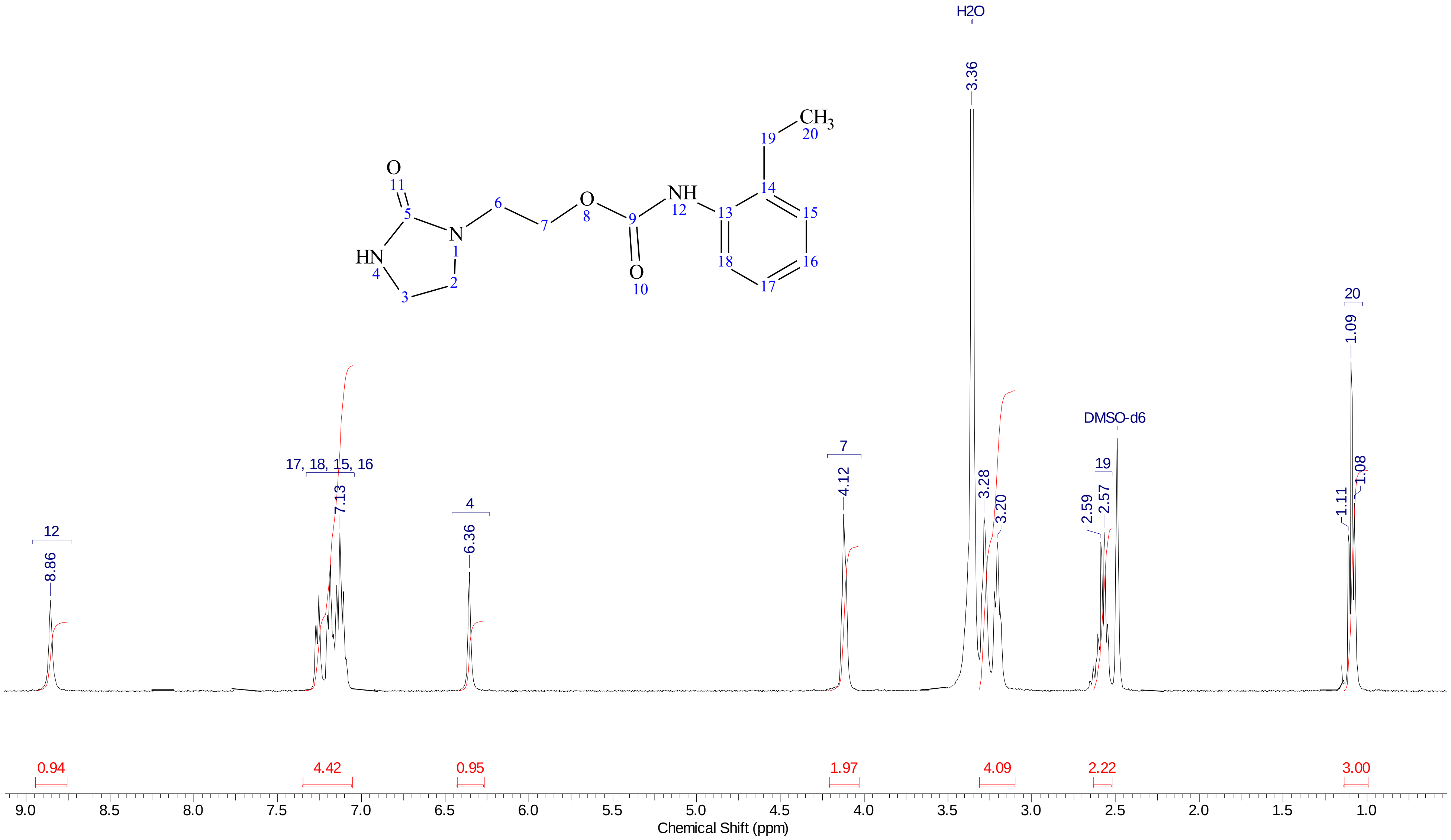

1H NMR spectrum of 2-(2-oxoimidazolidin-1-yl)ethyl-N-(2-ethylphenyl) carbamate, DMSO-d6, 400 MHz

2-(2-oxoimidazolidin-1-yl)ethyl-N-(4-methoxyphenyl) carbamate (9)

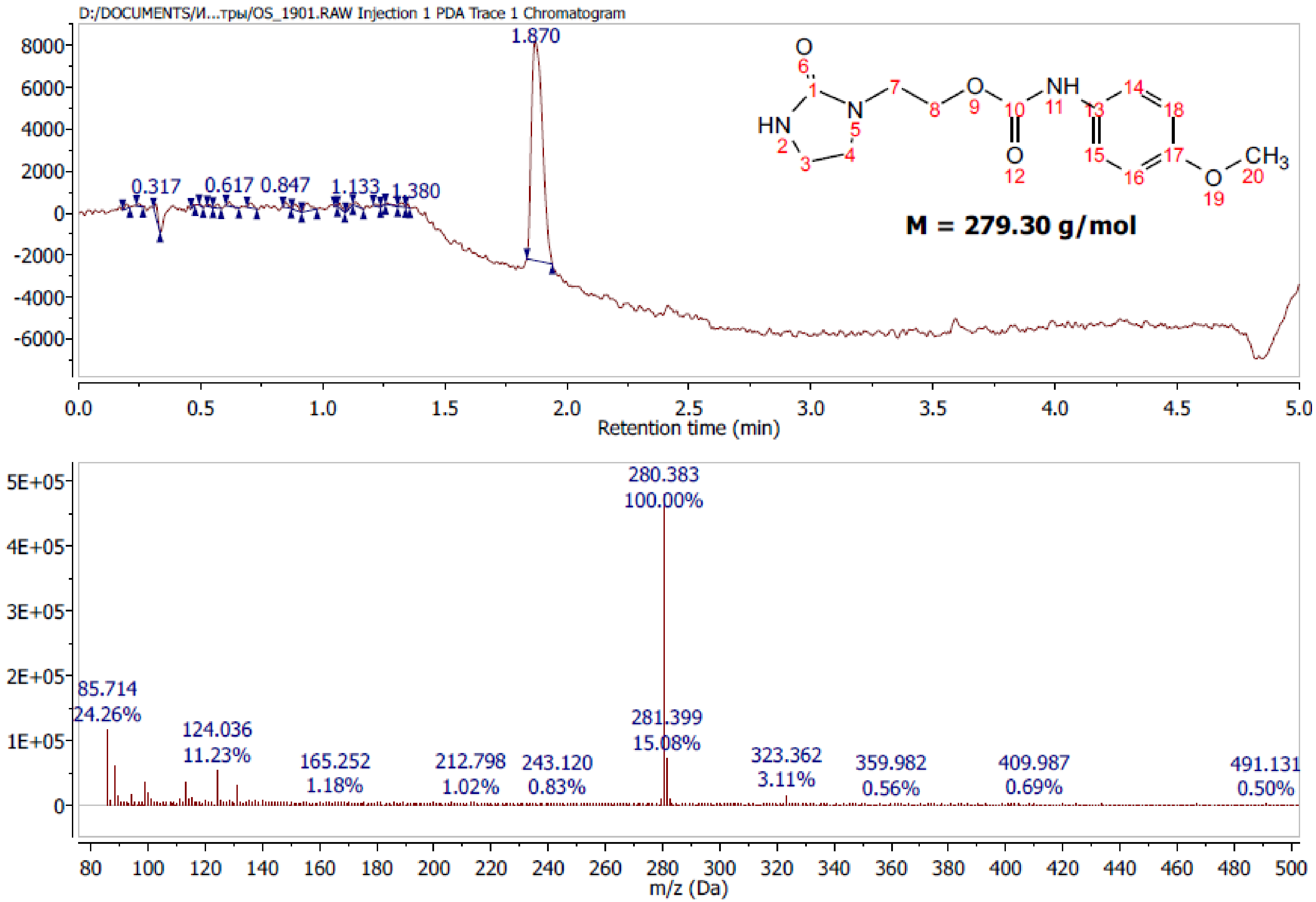

HPLC-MS spectrum of 2-(2-oxoimidazolidin-1-yl)ethyl-N-(4-methoxyphenyl) carbamate

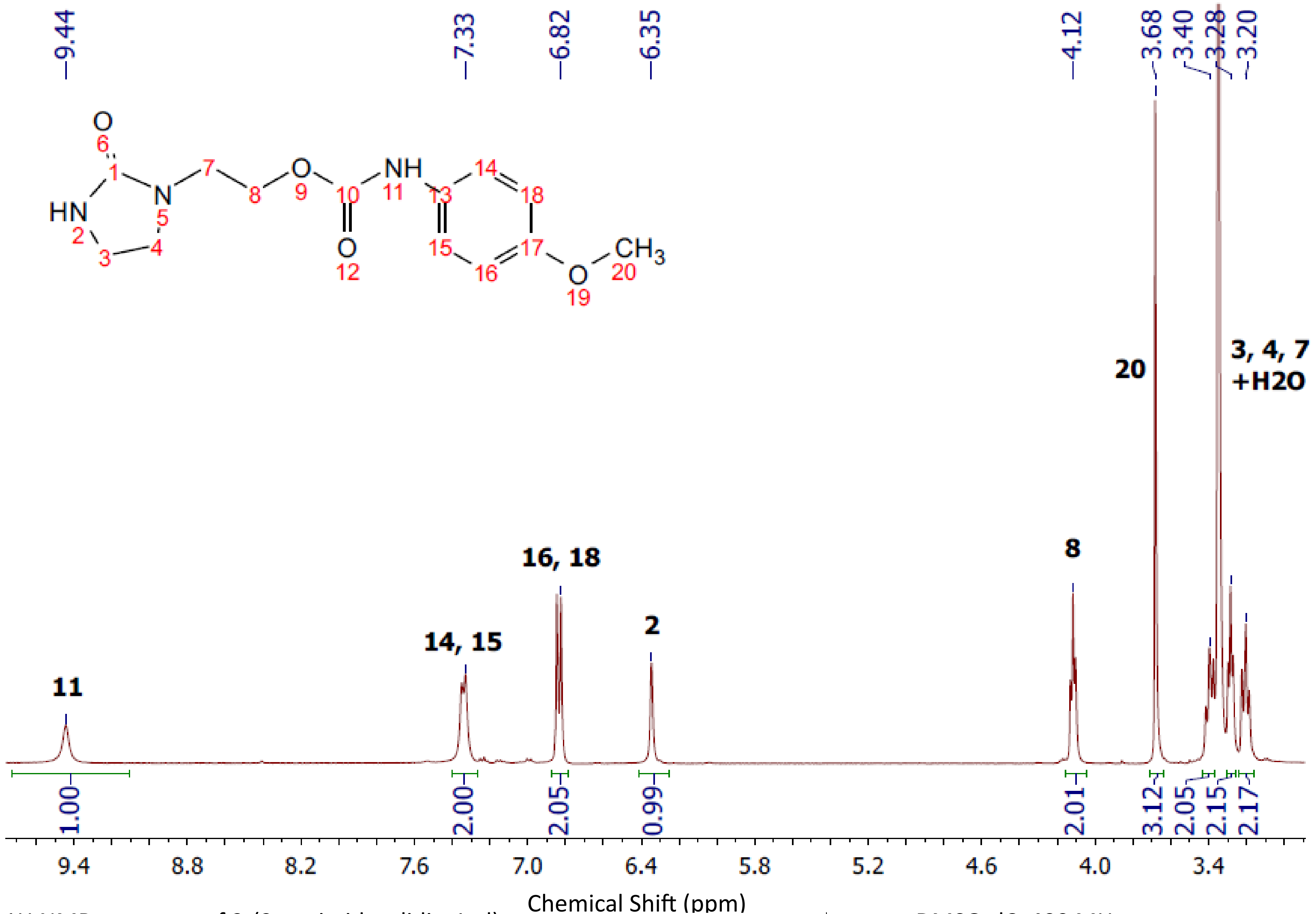

1H NMR spectrum of 2-(2-oxoimidazolidin-1-yl)ethyl-N-(4-methoxyphenyl) carbamate, DMSO-d6, 400 MHz

2-(2-oxoimidazolidin-1-yl)ethyl-N-(4-methoxycarbonylphenyl) carbamate (10)

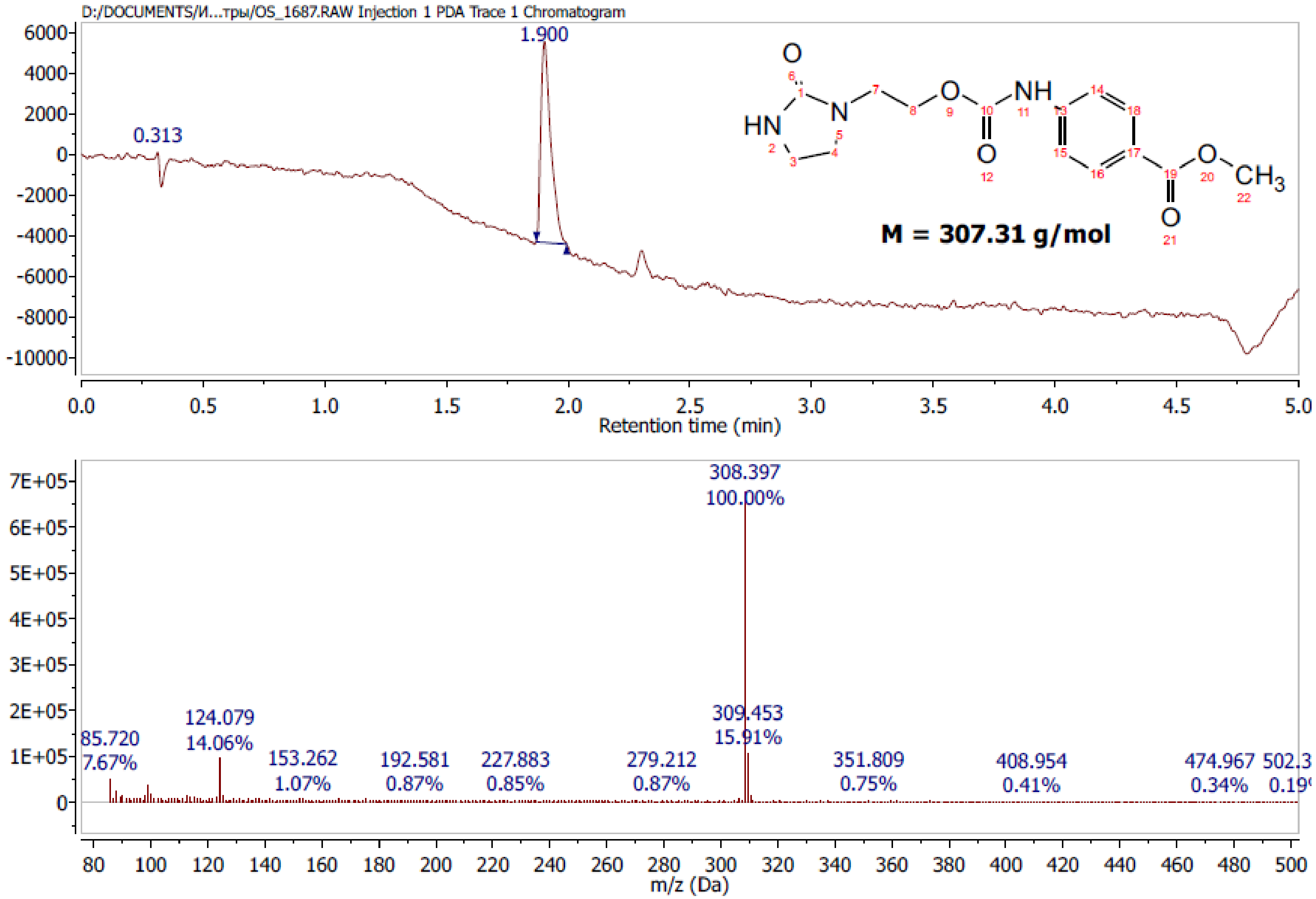

HPLC-MS spectrum of 2-(2-oxoimidazolidin-1-yl)ethyl-N-(4-methoxycarbonylphenyl) carbamate

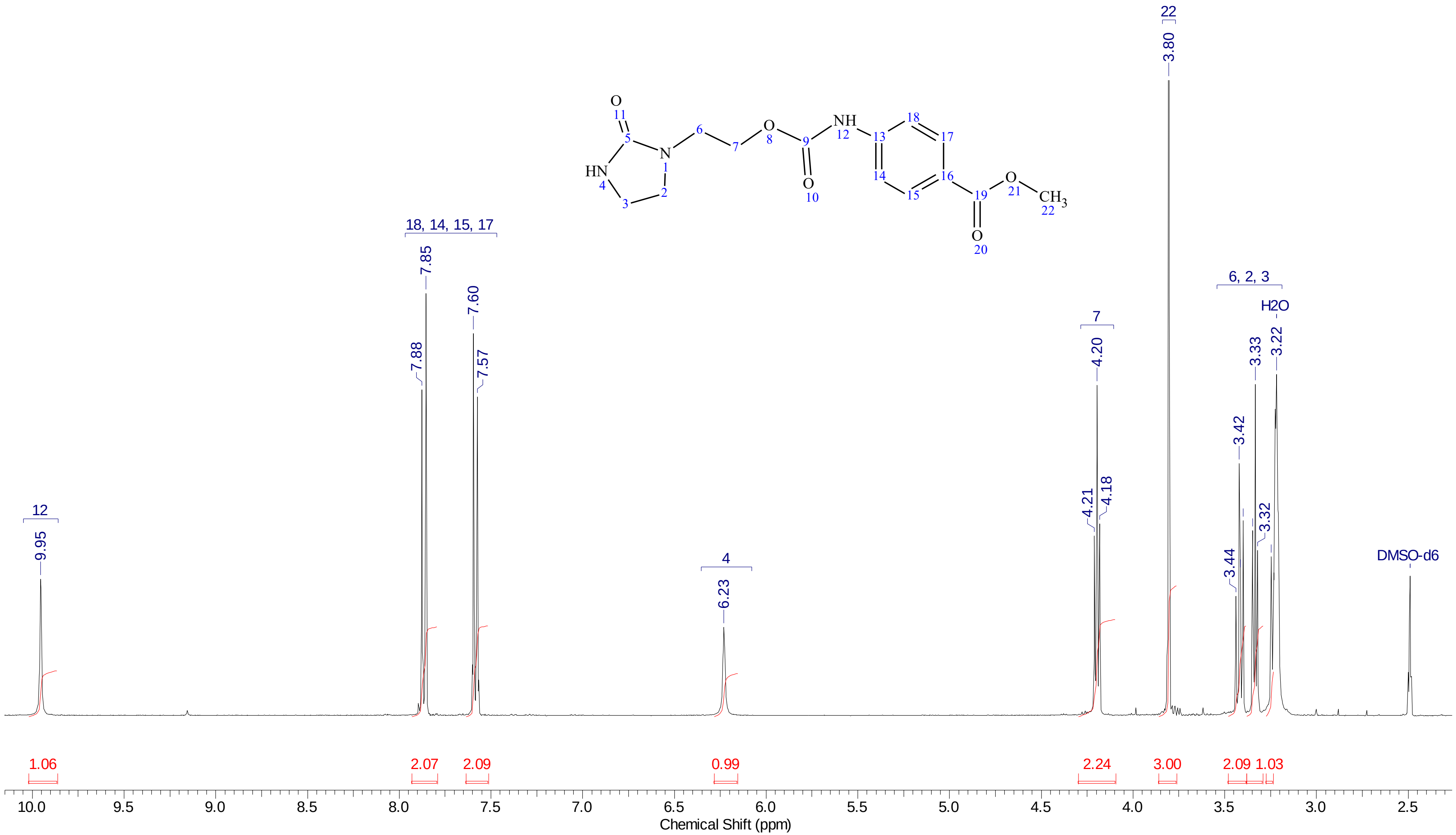

1H NMR spectrum of 2-(2-oxoimidazolidin-1-yl)ethyl-N-(4-methoxycarbonylphenyl) carbamate, DMSO-d6, 400 MHz
